# Tailored Monolayer Co‐Assembly for Enhanced Efficiency and Stability in Inverted Perovskite Solar Cells

**DOI:** 10.1002/advs.202521037

**Published:** 2026-01-04

**Authors:** Fen Xia, Shuwen Yang, Xiaolin Liu, Jing Zhang, Jun Yin, Jing Li, Zhaohui Wang, Song Tu, Binghui Wu, Nanfeng Zheng

**Affiliations:** ^1^ State Key Laboratory for Physical Chemistry of Solid Surfaces Collaborative Innovation Center of Chemistry for Energy Materials (iChEM) National & Local Joint Engineering Research Center of Preparation Technology of Nanomaterials College of Chemistry and Chemical Engineering, Pen‐Tung Sah Institute of Micro‐Nano Science and Technology Innovation Laboratory For Sciences and Technologies of Energy Materials of Fujian Province (IKKEM) Xiamen University Xiamen China

**Keywords:** self‐assembled monolayers, inverted perovskite solar cells, competitive co‐assembly, interfacial engineering

## Abstract

Self‐assembled monolayers (SAMs) are extensively employed as hole‐selective interlayers in inverted perovskite solar cells (PSCs), yet their incomplete coverage and poor molecular ordering often induce interfacial defects and exacerbate non‐radiative recombination. To overcome these limitations, we developed a bulky methylthio‐functionalized carbazole‐based SAM, (2‐(3,6‐bis(bis(4‐(methylthio)phenyl)amino)‐9*H*‐carbazol‐9‐yl)ethyl)phosphonic acid (S‐2PACz), and implemented a sequential co‐assembly strategy utilizing cysteine (Cys). In this tailored architecture, Cys is pre‐anchored via  carboxyl groups, followed by the competitive adsorption of S‐2PACz via phosphonic acid anchoring, yielding a mixed Cys‐S‐2PACz monolayer. The size complementarity and cooperative anchoring facilitated denser molecular packing and improved ordering, while synergistic methylthio and thiol functionalities jointly passivated interfacial defects. Consequently, this strategy enhanced perovskite crystallization, optimized energy‐level alignment, and facilitated efficient hole extraction. Ultimately, PSCs incorporating Cys‐S‐2PACz achieved higher power conversion efficiency, reduced recombination losses, and demonstrated superior thermal, operational, and outdoor stability compared with devices based on single‐component SAMs. This work establishes competitive co‐assembly as an effective interfacial engineering strategy, providing a scalable route to tailored SAM architectures for high‐performance and durable p–i–n PSCs.

## Graphical abstract

This work presents a co‐assembled monolayer, Cys‐S‐2PACz, constructed via sequential deposition. The size‐complementary pairing and competitive anchoring between Cys and S‐2PACz enable dense and highly ordered molecular packing. Meanwhile, the synergistic passivation from the methylthio and thiol groups effectively suppresses interfacial recombination, delivering enhanced efficiency and stability in inverted perovskite solar cells.

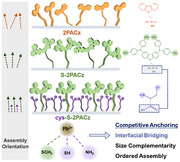

## 1 Introduction

Metal halide perovskites have emerged as leading candidates for next‐generation photovoltaics owing to their outstanding optoelectronic properties demonstrated over the past decade [1, 2, 3]. Among different device architectures, inverted perovskite solar cells (PSCs) are particularly attractive due to their fabrication scalability and operational stability. However, further advancement is hindered by the interfacial defects at the buried interface [4], which act as non‐radiative recombination centers and thereby limit power conversion efficiency (PCE) and long‐term reliability.

Self‐assembled monolayers (SAMs) have recently gained prominence as effective hole transport layers (HTLs) in inverted PSCs. By forming ordered molecular layers on transparent conductive oxides, SAMs regulate surface energy levels, passivate interfacial traps, and modulate perovskite crystallization [5, 6, 7, 8]. Yet, on rough metal oxide substrates, SAM formation is often incomplete due to limited solubility and insufficient chemical bond formation with the substrate, leading to heterogeneous coverage [9, 10, 11]. The buried interfaces, therefore remain defective and act as non‐radiative recombination centers, thereby limiting photovoltaic efficiency and operational stability [12].

To mitigate these limitations, various co‐assembly strategies have been explored. Blending molecules of similar anchoring groups, such as Me‐4PACz and MeO‐2PACz, enables energy‐level tuning and enhances thermal stability, but performance depends sensitively on precise molecular ratios [13, 14, 15]. Additive engineering offers an alternative, where imidazole derivatives prevent SAM aggregation [16] and 3‐mercaptopropionic acid disrupts 2PACz clusters [11]. Incorporating molecules with different anchoring groups, such as β‐guanidinopropionic acid with MeO‐2PACz, promotes multiple passivation pathways [17]. Furthermore, post‐assembly approaches employing bridging molecules like 5‐(9*H*‐carbazol‐9‐yl)isophthalic acid improve charge transport by facilitating π–π stacking interactions and Pb chelation at the interface [18]. Despite these advances, the roles of molecular interactions in the assembly process remain insufficiently clarified, making it challenging to realize dense and well‐ordered monolayers consistently.

In this work, we propose a sequential co‐assembly strategy leveraging steric complementarity to overcome these challenges. A small carboxylic acid molecule, cysteine hydrochloride (Cys·HCl), is pre‐anchored onto the substrate. Subsequently, a newly designed bulky phosphonic acid SAM, 2‐(3,6‐bis(bis(4‐(methylthio)phenyl)amino)‐9*H*‐carbazol‐9‐yl)ethyl)phosphonic acid (S‐2PACz), is introduced through a competitive assembly process. Size complementarity and cooperative anchoring modulate interfacial morphology and molecular orientation, while methylthio and thiol groups provide synergistic defect passivation and interfacial coupling. This strategy achieves dense, well‐ordered SAM layers, reduces interfacial recombination, and substantially improves device efficiency and stability. Our results demonstrate a rational pathway for tailoring SAM assembly, providing a robust strategy to enhance the performance and durability of inverted PSCs.

### 1.1 Design and Co‐Assembly Strategy

**FIGURE 1:**
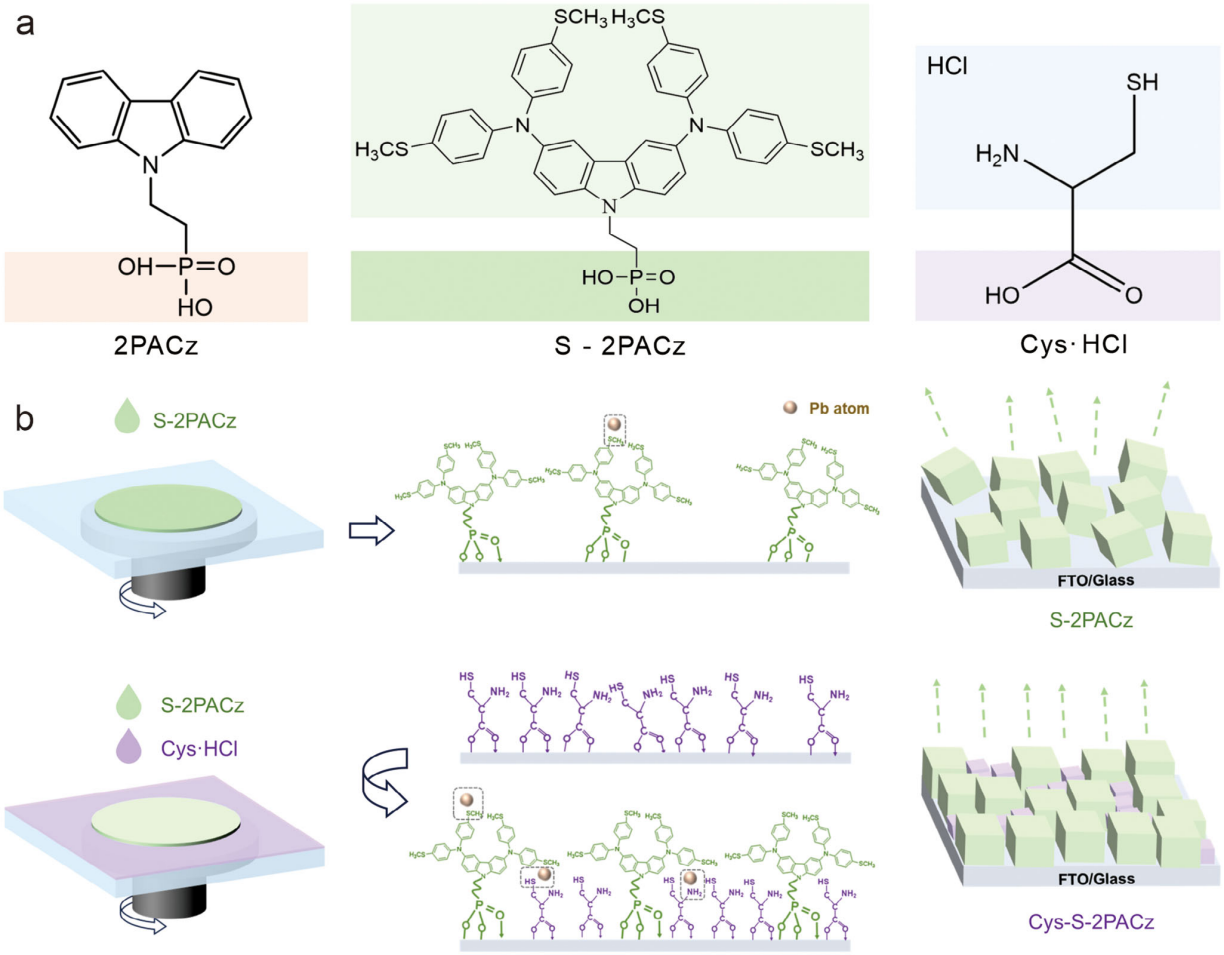
Molecular design and interfacial engineering strategy. (a) Molecular structures of 2PACz, S‐2PACz, and Cys·HCl. (b) Schematic illustration of the comparative assembly behaviors of S‐2PACz and Cys‐S‐2PACz on the FTO substrates.

The synthetic route of the hole‐selective SAM molecule, S‐2PACz, is illustrated in Figure S1. The chemical structures of the intermediates and the final compound were verified by ^1^H and ^13^C NMR spectroscopy (Figures S2–S11). Compared to conventional 2PACz, S‐2PACz features an extended conjugated backbone composed of electron‐rich diphenylamine units functionalized with methylthio groups (Figure 1a). This design is expected to enhance charge transport [19], while the methylthio terminals provide stronger interfacial binding through Lewis base character and Pb─S coordination [20]. Density functional theory (DFT) calculations suggest delocalized HOMO distribution along the carbazole backbone and increased electron density at the methylthio groups (Figure S12), which may enhance the interactions with Pb^2+^ from perovskite layers [21]. To evaluate the energy level alignment, cyclic voltammetry and ultraviolet photoelectron spectroscopy (UPS) measurements were performed, yielding HOMO levels of −5.21 and −5.46 eV, respectively, while UV–vis spectra indicated an optical bandgap of 3.51 eV, showing good alignment with the valence band of the perovskite (Figures S13–S16). Thermal analysis determined a glass transition temperature of 133°C and decomposition temperature of 240°C (corresponding to 5 % weight loss), as shown in Figures S17 and S18, suggesting sufficient thermal stability for the fabrication of PSCs.

To further improve SAM assembly quality, Cys·HCl, a small carboxylic acid molecule with a thiol group, was introduced to co‐assemble with S‐2PACz. The molecular structure is shown in Figure 1a. The strategy employed sequential spin‐coating of Cys·HCl and S‐2PACz onto FTO substrates, enabling Cys pre‐anchoring via its carboxyl group before partial replacement by the phosphonic acid‐based SAM (Figure 1b). Since carboxylic acids exhibit lower binding affinity than phosphonic acids [22, 23], Cys provides initial attachment sites, while its short linear chain minimizes steric hindrance, facilitating close co‐assembly with S‐2PACz. This competitive assembly, driven by differences in anchoring strength and molecular size complementarity, may promote enhanced molecular order and packing density. This co‐assembled two‐component SAM (Cys‐S‐2PACz) is designed to maximize interfacial coverage and optimize contact with the buried perovskite layer.

To probe potential cooperative interactions of S‐2PACz and Cys with Pb^2+^ that strengthen interfacial bonding, spectroscopic analyses were conducted. Raman spectroscopy of S‐2PACz mixed with PbI_2_ revealed emerging peaks at 165 and 216 cm^−1^ (Figure S19), attributed to Pb─S bonding [24]. For Cys, the terminal –SH groups would coordinate with undercoordinated Pb^2+^ sites, which may modulate crystallization and improve film morphology [25]. Fourier transform infrared spectra showed that the S–H stretching vibration at 2,552 cm^−1^ weakened significantly upon PbI_2_ addition, indicating thiol−Pb bonding (Figure S20) [26, 27]. Together, these results indicate that co‐assembly leads to the formation of synergistic interfacial bridging via the coordination of S‐2PACz and Cys with Pb^2+^.

### 1.2 Competitive Co‐Assembly of SAMs

#### 1.2.1 Co‐Assembly on FTO

**FIGURE 2:**
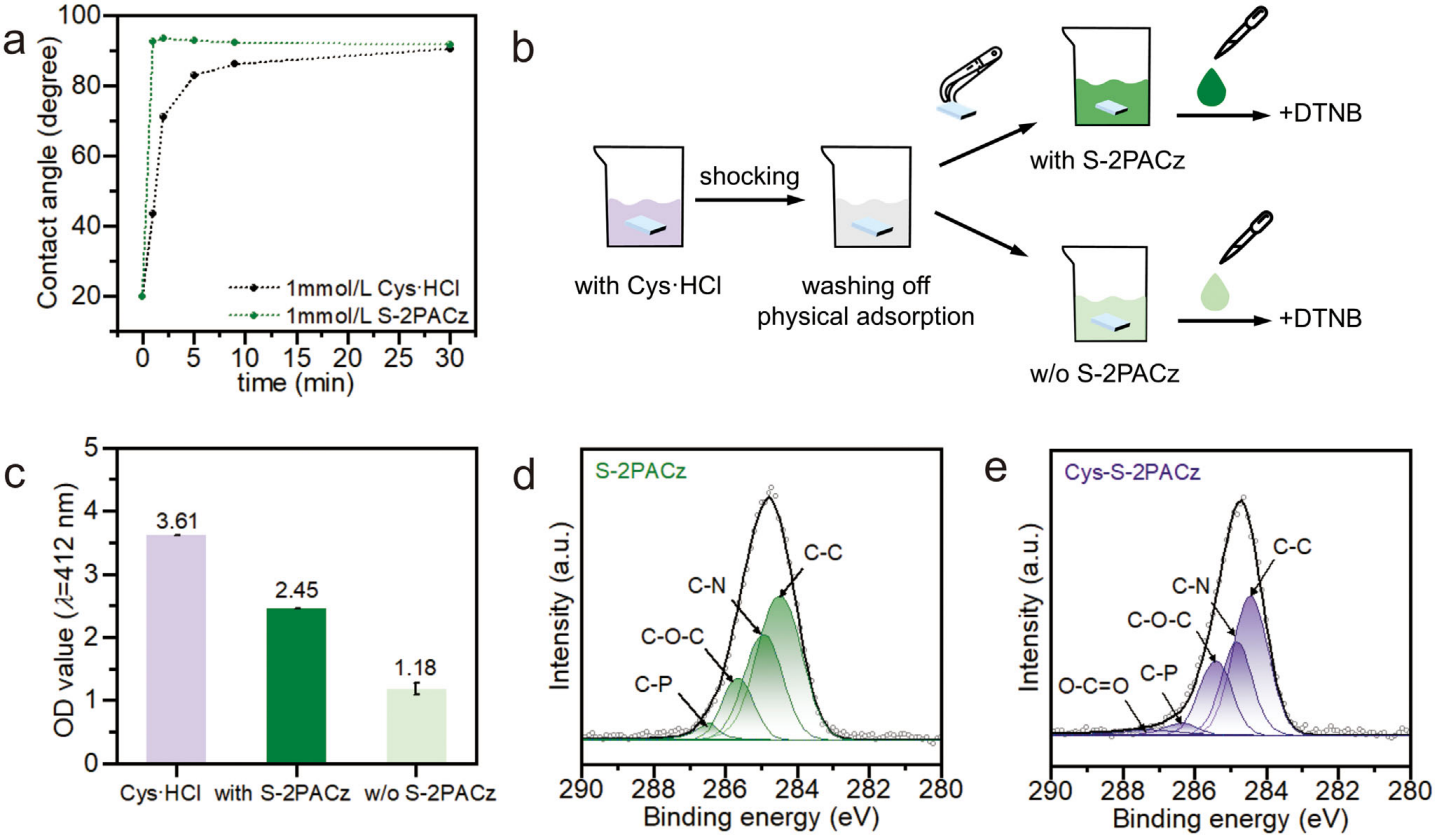
Competitive co‐assembly of S‐2PACz and Cys·HCl on FTO. (a) Time‐dependent adsorption of Cys·HCl and S‐2PACz on FTO monitored by static contact angles. (b) Schematic illustration of thiol detection by the Ellman assay using DTNB (5,5′‐dithiobis(2‐nitrobenzoic acid)) as the chromogenic reagent. (c) OD values at 412 nm for the corresponding samples. (d) XPS C 1s spectrum of the S‐2PACz film. (e) XPS C 1s spectrum of the Cys‐S‐2PACz film.

To elucidate the competitive co‐assembly mechanism of S‐2PACz with Cys·HCl on FTO substrates, both indirect and direct evidence were collected. Indirect evidence was obtained from time‐dependent static contact angle measurements, which monitored the single‐component adsorption of these two molecules at equal molar concentrations (Figure 2a). S‐2PACz reached saturation within 1 min, whereas Cys·HCl required more than 10 min, suggesting that S‐2PACz exhibited stronger adsorption on the FTO surface. For direct confirmation, FTO substrates were first immersed in Cys·HCl solution, rinsed to remove weakly bound molecules, and then transferred into solutions with and without S‐2PACz, respectively. The concentration of desorbed Cys was quantified by the Ellman assay, detecting free thiol groups through optical density (OD) at 412 nm (Figure 2b) [28]. A higher OD value observed after introducing S‐2PACz confirmed that pre‐adsorbed Cys was displaced, consistent with a competitive displacement mechanism (Figure 2c). Finally, X‐ray photoelectron spectroscopy (XPS) further validated the co‐assembly: C 1s peaks at 286.4 eV (C─P), and 287.3 eV (O─C═O) confirmed the simultaneous presence of both molecules on the FTO surface (Figure 2d,e) [13]. Collectively, these results establish that S‐2PACz partially replaces pre‐anchored Cys, demonstrating a competitive co‐assembly mechanism.

#### 1.2.2 Surface Coverage of SAMs

After confirming the competitive co‐adsorption mechanism between S‐2PACz and Cys·HCl on FTO, the quality of the assembled layer was evaluated, as compact SAM coverage is crucial for efficient hole extraction in PSCs. The surface packing density of adsorbed molecules on conductive electrodes is typically quantified by cyclic voltammetry, where the anodic peak current is linearly proportional to the scan rate according to the following relationship [29]:

$$i_{p}=\frac{n^{2}F^{2}}{4RTN_{A}}\;A\Gamma^{*}v$$

Here, *i_p_* (A) represents the anodic peak current, *v* (V s^−1^) is the voltage scan rate, *n* is the number of electrons transferred, *F* (96485.33 C mol^−1^) is the Faraday constant, *R* (8.314 J K^−1^ mol^−1^) is the universal gas constant, *T* (K) is the absolute temperature, *N_A_* (6.022 × 10^23^ mol^−1^) is the Avogadro constant, *A* (cm^2^) is the electrode surface area, and Γ*(molecules cm^−2^) is the surface density. The surface coverage was extracted from the slope of *i_p_* vs. *v* plot (Figure S21). The measured densities were 1.33 ± 0.24 × 10^14^ and 1.78 ± 0.12 × 10^14^ molecules cm^−2^ for S‐2PACz and Cys‐S‐2PACz, respectively. The higher coverage and reduced standard deviation of the Cys‐S‐2PACz layer are attributed to cooperative adsorption and molecular size complementarity, resulting in a more densely packed molecular assembly on the FTO surface.

#### 1.2.3 Molecular Ordering of SAMs

**FIGURE 3:**
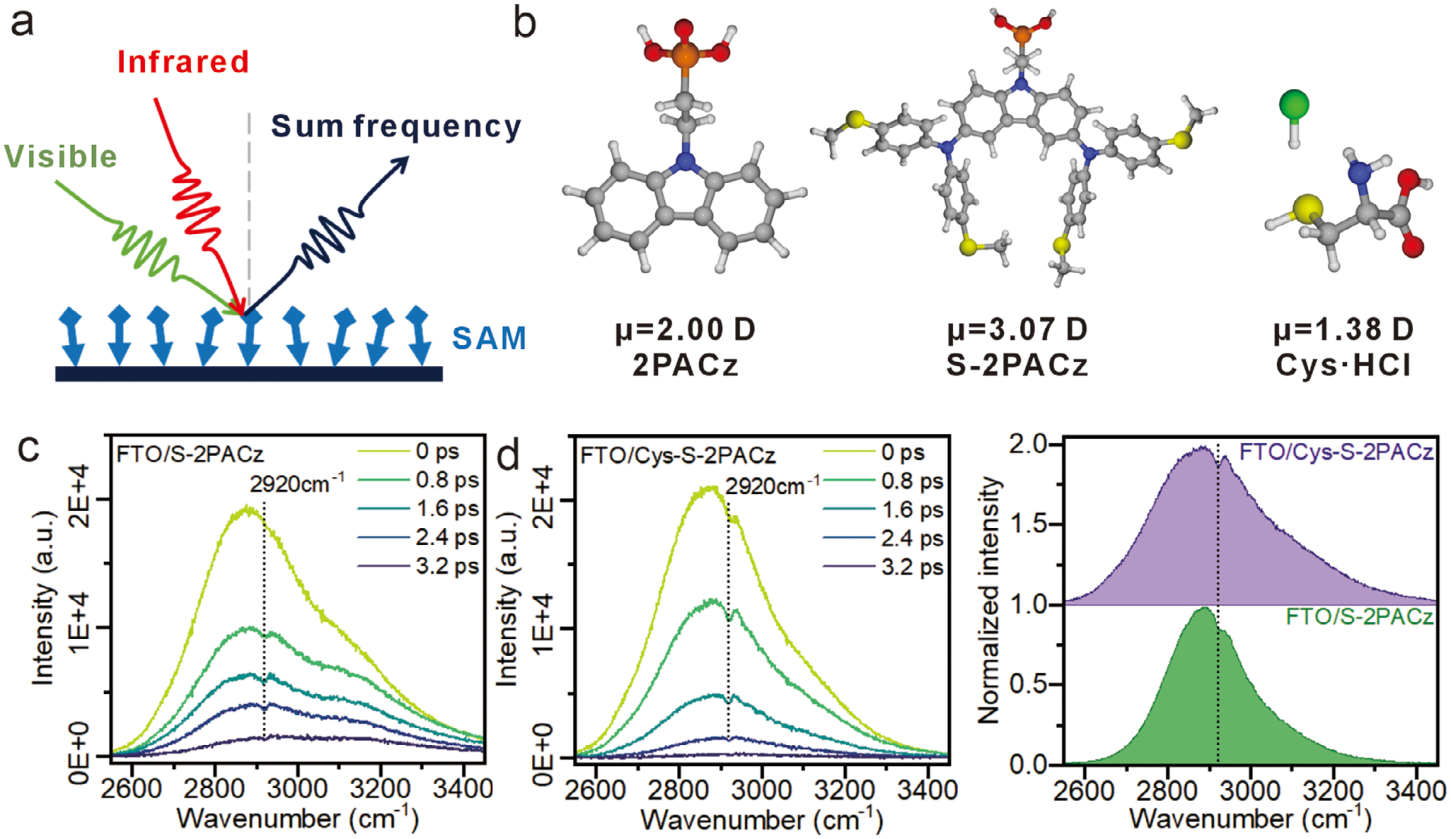
Molecular ordering of SAMs revealed by SFG spectroscopy. (a) Schematic illustration of the working principle of SFG measurements. (b) Calculated dipole moments of 2PACz, S‐2PACz, and Cys·HCl. (c) BB‐SFG spectra of the FTO/S‐2PACz sample. (d) BB‐SFG spectra of the FTO/Cys–S‐2PACz sample. (e) Normalized BB‐SFG spectral intensity at 0.8 ps.

Following confirmation of enhanced packing density, the molecular ordering of the Cys‐S‐2PACz monolayer was investigated to further assess its interfacial structural quality. Sum‐frequency generation (SFG) vibrational spectroscopy, a second‐order nonlinear technique inherently sensitive to surfaces, was used to probe the interfacial structure (Figure 3a). SFG arises from the coherent mixing of infrared and visible beams at a noncentrosymmetric interface to generate a new frequency. Its intensity directly correlates with the net ordering of dipole‐active vibrational modes oriented perpendicular to the substrate [30]. To rationalize the signals, DFT calculations of dipole moments were performed, revealing a larger dipole moment for S‐2PACz (3.07 D) compared to that of 2PACz (2.00 D) and Cys·HCl (1.38 D) (Figure 3b). Broadband SFG (BB‐SFG) measurements showed negligible spectral features for FTO, FTO/2PACz, and FTO/Cys·HCl, which may be attributed to substrate roughness and limited dipole strength (Figure S22). In contrast, FTO/S‐2PACz exhibited a weak CH_2_ asymmetric stretch near 2,920 cm^−1^, indicating partial chain ordering (Figure 3c). Strikingly, FTO/Cys‐S‐2PACz displayed a much stronger CH_2_ signal, consistent with improved molecular alignment and higher packing density, which enhances intermolecular packing (Figure 3d,e). These results demonstrate that size complementarity and cooperative anchoring between Cys·HCl and S‐2PACz reduce orientation dispersion, thereby yielding more ordered SAMs. Such ordering is expected to reduce trap‐assisted recombination and facilitate energy‐level alignment, ultimately improving device performance.

### 1.3 Film Morphology and Electronic Properties

**FIGURE 4:**
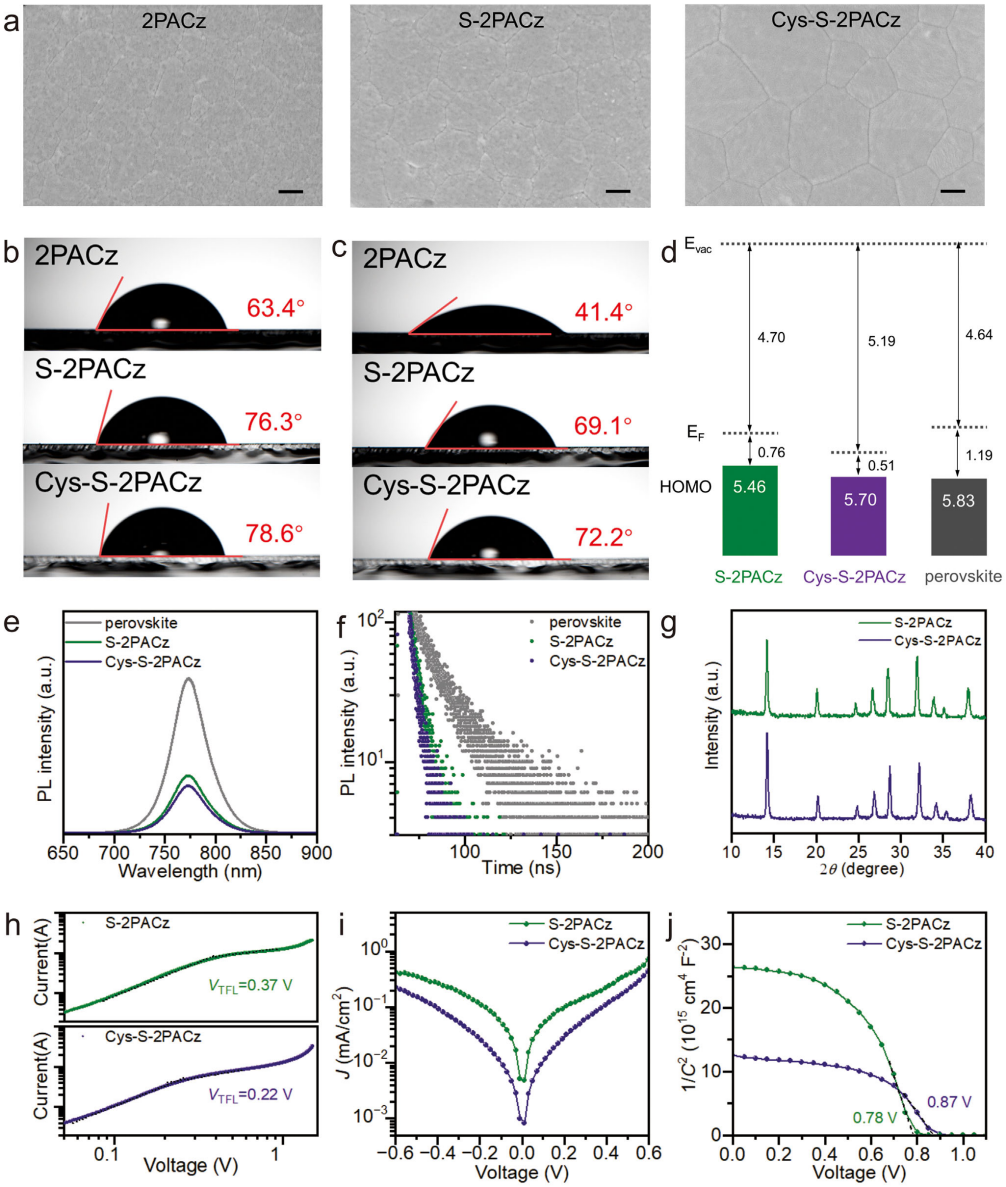
Morphology and electronic properties of perovskite films and devices on SAM‐modified substrates. (a) SEM images of perovskite films peeled off from ITO substrates modified with 2PACz, S‐2PACz, and Cys‐S‐2PACz SAMs (scale bar: 300 nm). (b, c) Water contact angles of FTO substrates modified with different SAMs b) at 0 min and c) after 6 min. (d) Schematic diagram of energy‐level alignment. (e) Steady‐state PL spectra. (f) TRPL spectra. (g) XRD patterns of perovskite films deposited on S‐2PACz and Cys‐S‐2PACz. (h) SCLC measurements of the hole‐only devices. (i) Dark *J–V* curves. (j) Mott–Schottky plots.

To assess the effects of the densely ordered Cys‐S‐2PACz co‐assembly on perovskite films and device‐relevant interfaces, comprehensive morphological and spectroscopic characterizations were performed. Scanning electron microscopy (SEM) revealed that perovskite films grown on Cys‐S‐2PACz exhibited larger grains than those deposited on 2PACz or S‐2PACz (Figure S23). To facilitate clear visualization of the buried interface, a smoother ITO substrate was employed instead of FTO. The differences were more pronounced at the perovskite bottom surface, where the film deposited on Cys‐S‐2PACz exhibited a continuous morphology, in contrast to the irregular boundaries and discontinuities observed on the single‐component SAM (Figure 4a). Static water contact angle measurements showed values of 63.4°, 76.3°, and 78.6° for 2PACz, S‐2PACz, and Cys‐S‐2PACz, respectively (Figure 4b) [13, 31]. Moreover, Cys‐S‐2PACz maintained the largest contact angle over 6 min (Figure 4c), indicating more compact and stable coverage [32]. UPS revealed that the Cys‐S‐2PACz layer possessed a deeper HOMO level (−5.70 eV) than S‐2PACz (−5.46 eV), yielding a smaller offset with the perovskite valence band (−5.83 eV) (Figures 4d and S24). This improved energetic alignment minimizes the barrier for hole extraction [33].

To further evaluate hole extraction ability, steady‐state photoluminescence (PL) and time‐resolved photoluminescence (TRPL) measurements were performed (Figure 4e,f; Table S1). Perovskite films on the Cys‐S‐2PACz substrate exhibited weaker PL intensity and shorter carrier lifetimes, indicating more efficient interfacial hole extraction. X‐ray diffraction (XRD) patterns further showed enhanced crystallinity for perovskite films grown on the Cys‐S‐2PACz substrate (Figure 4g), with the full width at half maximum of the (100) diffraction peak decreasing from 0.223° (control) to 0.163°. Defect characteristics were probed by space‐charge‐limited current (SCLC) measurements. The trap‐filled limit voltage (*V*_TFL_) decreased from 0.37 V for S‐2PACz to 0.22 V for Cys‐S‐2PACz (Figure 4h), corresponding to lower trap density. Consistently, dark current analysis revealed reduced leakage current in Cys‐S‐2PACz‐based devices (Figure 4i). Mott–Schottky plots showed a higher built‐in potential for Cys‐S‐2PACz (0.87 V) compared to S‐2PACz (0.78 V), indicating a stronger driving force for charge separation (Figure 4j). Overall, these morphological, optical, and electronic characterizations confirmed that Cys‐S‐2PACz was able to improve interfacial quality, reduce trap density, and strengthen charge extraction, leading to enhanced device performance.

### 1.4 Device Performance and Operational Stability

**FIGURE 5:**
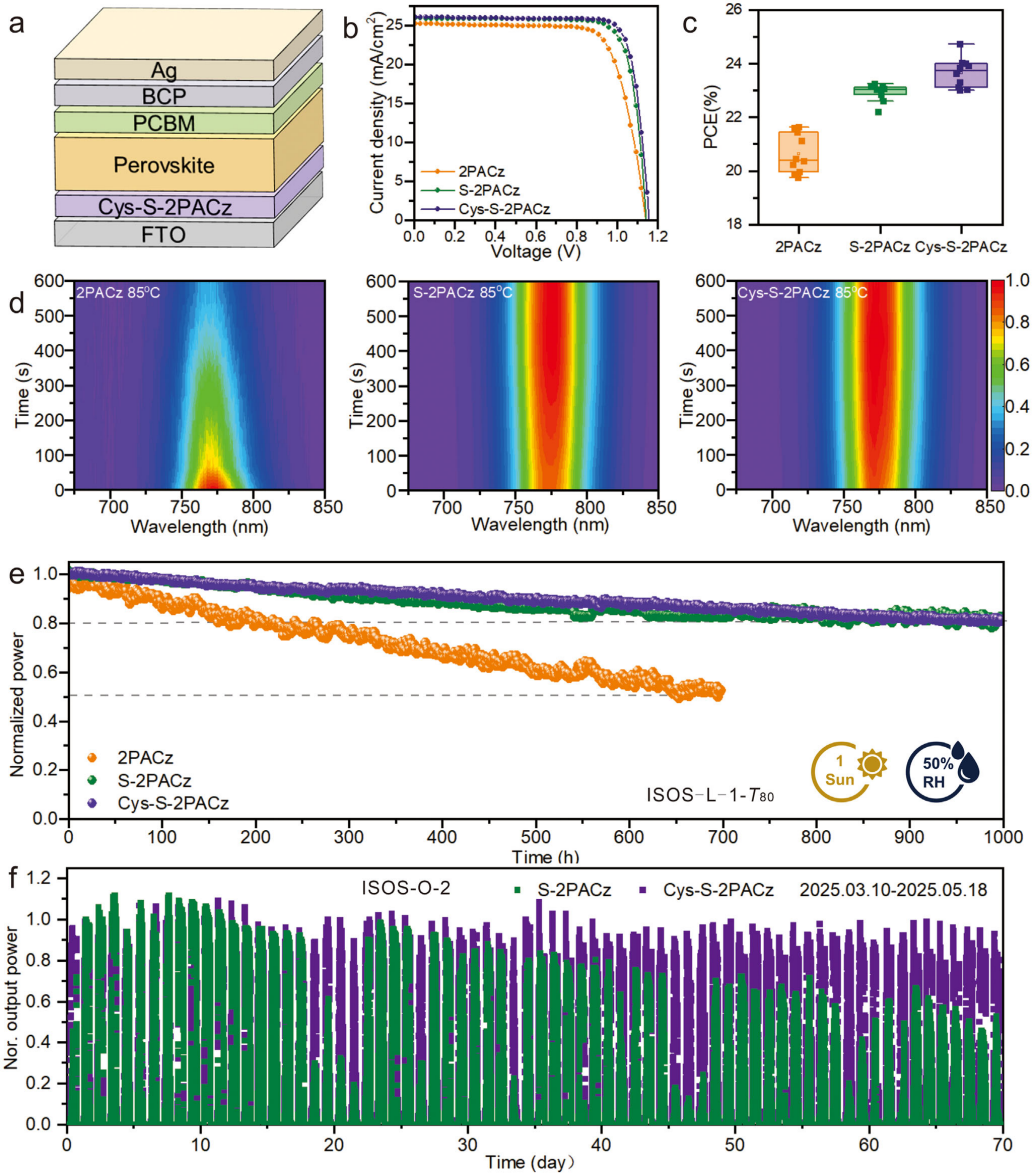
Device performance and operational stability of inverted PSCs. (a) Schematic illustration of the device structure with Cys‐S‐2PACz. (b) *J‐V* curves of champion devices based on different SAMs. (c) Statistical distribution of PCEs. (d) In situ evolution of PL spectra of perovskite films on 2PACz, S‐2PACz, and Cys‐S‐2PACz under continuous heating at 85 °C in air. (e) Long‐term operational stability of encapsulated devices under simulated one‐sun illumination. (f) Outdoor performance comparison between S‐2PACz and Cys‐S‐2PACz modules.

Building on the improved interfacial properties, the co‐assembly strategy was applied to fabricate inverted PSCs with a device architecture of FTO/SAM/perovskite/PCBM/BCP/Ag (Figure 5a). The current density–voltage (*J–V*) characteristics are shown in Figure 5b, with detailed photovoltaic parameters summarized in Table S2. The champion Cys‐S‐2PACz‐based device achieved a champion PCE of 24.72% (*V*_OC_ = 1.15 V, *J*_SC_ = 26.09 mA cm^−2^, FF = 82.10%), higher than the best devices based on 2PACz (21.64%) and S‐2PACz (23.84%). External quantum efficiency (EQE) spectra confirmed that the integrated *J*_SC_ value was consistent with the *J–V* result (Figure S25). Furthermore, Cys‐S‐2PACz‐based devices exhibited the highest average performance metrics across multiple samples (Figure 5c; Figure S26), confirming the reproducibility of efficiency improvements.

The stability of the devices was then evaluated under thermal and humid stress conditions. Using an in situ PL system, unencapsulated SAMs/perovskite films were heated at 85°C under ambient conditions with a relative humidity (RH) of 50%±10%. The PL intensity of the perovskite film on 2PACz decreased to below 80% within 200 s, whereas the perovskite films on S‐2PACz and Cys‐S‐2PACz retained stable signals for 600 s (Figure 5d). This improved stability was attributed to hydrophobic surfaces, effective defect passivation, and denser SAM coverage, suppressing moisture ingress and non‐radiative recombination.

To further validate the practicality, perovskite solar modules (PSMs) were fabricated using the co‐assembly strategy. The 6 × 6 cm^2^ PSMs with 22.45 cm^2^ active area and 95.79% geometric fill factor (Figures S27 and S28) achieved a champion PCE of 17.54% for Cys‐S‐2PACz, surpassing 2PACz (15.55%) and S‐2PACz (17.10%) (Figure S29 and Table S3). Operational stability, evaluated according to the ISOS‐L‐1 protocol under continuous AM 1.5G illumination with maximum power point (MPP) tracking (50%±10% RH), showed that Cys‐S‐2PACz and S‐2PACz modules retained 80% of their initial output after 1000 h, whereas the control device degraded to about 50% within 700 h (Figure 5e). Outdoor stability was further assessed under the ISOS‐O‐2 protocol using a custom‐built solar‐tracking system [34, 35], with device surface temperatures reaching up to 65°C (Figure S30). After 18 days, the Cys‐S‐2PACz device still maintained stable power output, whereas the S‐2PACz device exhibited a noticeable decline (Figure 5f). These results collectively demonstrate the enhanced efficiency and stability of the co‐assembly strategy, confirming its viability for practical photovoltaic applications.

## 2 Conclusion

This work demonstrates a cooperative assembly strategy that enables interfacial engineering for high‐performance perovskite solar cells. Sequential deposition allowed Cys to initially anchor through carboxyl groups, followed by S‐2PACz binding via phosphonic acid groups, establishing competitive adsorption between functional moieties. This process promoted dense and ordered packing of the co‐assembled SAMs. Size complementarity improved molecular organization, while methylthio and thiol groups provided synergistic passivation of the buried perovskite interface. As a result, the co‐assembled SAM exhibited enhanced interfacial quality, suppressed trap states, and facilitated efficient hole transport. Devices based on this strategy delivered improved efficiency, thermal durability, and outdoor operational stability compared to single‐component SAMs. Overall, the competitive co‐assembly mechanism offers a robust and scalable pathway for constructing stable interfaces in perovskite photovoltaics, advancing both performance and long‐term reliability.

## Conflicts of Interest

The authors declare no conflicts of interest.

## Supporting information




**Supporting File**: advs73581‐sup‐0001‐SuppMat.docx

## Data Availability

The data that support the findings of this study are available from the corresponding author upon reasonable request.
