# Supplementary material for: Tailored Monolayer Co‐Assembly for Enhanced Efficiency and Stability in Inverted Perovskite Solar Cells
Source: Adv Sci (Weinh). 2026 Jan 4;13(14):e21037. doi: 10.1002/advs.202521037 (PMC12970287; doi:10.1002/advs.202521037)
Supplement: Supplementary file 1 — Supporting File: advs73581‐sup‐0001‐SuppMat.docx [file ADVS-13-e21037-s001.docx]

Supporting Information

Tailored Monolayer Co-Assembly for Enhanced Efficiency and Stability in Inverted Perovskite Solar Cells

Fen Xia, Shuwen Yang, Xiaolin Liu, Jing Zhang, Jun Yin, Jing Li, Zhaohui Wang,* Song Tu,* Binghui Wu,* Nanfeng Zheng

Materials.

All chemicals and reagents were used as received without further purification. Lead iodide (PbI_2_), lead bromide (PbBr_2_), lead chloride (PbCl_2_), methylamine hydrochloride (MACl), methylammonium iodide (MAI), methylammonium bromide (MABr), cesium iodide (CsI), rubidium iodide (RbI), 2,9-dimethyl-4,7-diphenyl-1,10-phenanthroline (BCP) , and C_60_ were purchased from Xi’an Yuri Solar Co., Ltd. Cysteine hydrochloride was purchased from Shanghai Dibai Biotechnology Co., Ltd. Formamidinium iodide (FAI) was obtained from Greatcell Solar Materials. *N, N*-Dimethylformamide (DMF), dimethyl sulfoxide (DMSO), isopropanol (IPA), ethanol, ethyl acetate (EA), chlorobenzene (CB), and *N*-methylpyrrolidone (NMP) were purchased from Sigma-Aldrich. Ethylenediammonium diiodide (EDAI_2_, 99.99%) and (2-(9H-carbazol-9-yl) ethyl) phosphonic acid (2PACz) was obtained from Tokyo Chemical Industry Co., Ltd. (TCI). 1-Butyl-3-methylimidazolium tetrafluoroborate (BMIMBF_4_) was purchased from Macklin Biochemical Co., Ltd. [6,6]-Phenyl-C_61_-butyric acid methyl ester (PC_61_BM) was purchased from Advanced Election Technology Co., Ltd. All other solvents were purchased from Sinopharm Chemical Reagent Co., Ltd.

Characterization.

The ^1^H NMR, ^13^C NMR, and ^31^P NMR spectra were recorded on a Bruker 400 MHz spectrometer, using deuterated chloroform (CDCl_3_) or deuterated dimethyl sulfoxide (DMSO-*d*_6_) as solvents, depending on the solubility of the compounds. High-resolution mass spectra (HRMS) were recorded on an Agilent QTOF 6550 mass spectrometer. Ultraviolet photoelectron spectroscopy (UPS) was executed using a PHI 5000 VersaProbe III with He I source (21.22 eV) under a negative bias of 5.0 V. Ultraviolet–visible (UV–vis) absorption spectra were recorded using a Shimadzu UV-2600 spectrophotometer. Differential scanning calorimetry (DSC) was carried out using a Diamond DSC instrument (PerkinElmer). The measurements were performed under a nitrogen (N_2_) atmosphere at a heating rate of 10 °C min^-1^, using aluminum crucibles as both sample holders and references. Thermogravimetric analysis (TGA) was performed on a TGA/SDTA851E instrument (Mettler Toledo). Prior to measurement, the samples were vacuum-dried at 60 °C for 12 h. TGA was conducted under a nitrogen (N_2_) atmosphere with a heating rate of 10 °C min^-1^. Raman spectra were collected using a LabRAM HR Evolution (Horiba) with a 532 nm laser excitation. Fourier transform infrared (FTIR) spectra were recorded under vacuum on a Bruker Vertex 70V spectrophotometer. Water contact measurements were conducted using a DSA-20 video optical contact angle measuring instrument with 1 µL drops of ultrapure water. X-ray photoelectron spectroscopy (XPS) measurements were recorded on a Thermo-Fisher ESCA-LAB 250Xi system employing monochromatized Al Kα radiation under the pressure of 5.0$\times$10^-7^ Pa. Field-emission scanning electron microscope (SEM, Zeiss GeminiSEM500) was employed to observe the morphology. X-ray diffraction (XRD) patterns were recorded using a Rigaku RINT-2500 diffractometer equipped with a Cu Kα radiation source (λ = 1.5406 Å). Steady-state photoluminescence (PL), and time-resolved photoluminescence (TRPL) were measured using an FLS1000 spectrometer (Edinburgh Instruments). In-situ PL spectra of HTL-perovskite films were recorded during aging on a hotplate at 85 °C in ambient air (50 ± 10% RH) under 375 nm laser excitation (0.3 W). PL spectra were automatically collected for 10 min.

All electrochemical characterizations, including cyclic voltammetry (CV), space-charge-limited current (SCLC) measurements and Mott-Schottky analysis, were conducted using a CHI760E electrochemical workstation. HOMO/LUMO energy levels were calculated using the equation: ^[S1]^

$\text{E}_{\text{HOMO}}\text{=-5.1-(}\text{E}_{\text{OX}}\text{-}\text{E}_{\text{1/2(Fc/}\text{Fc}^{\text{+}}\text{）}}\text{)}$ (1)

$\text{E}_{\text{LUMO}}\text{=}\text{E}_{\text{HOMO}}\text{+}\text{E}_{\text{g}}^{\text{opt}}$ (2)

CV measurements were carried out using a three-electrode system. A glassy carbon electrode was used as the working electrode, a platinum sheet as the counter electrode, and an Ag/AgCl electrode (saturated KCl solution) as the reference electrode. Prior to measurements, the surface of the glassy carbon electrode was polished with α-alumina powder. The electrode was then sequentially rinsed with deionized water, anhydrous ethanol, and dichloromethane (DCM) to remove surface contaminants that could affect the measurements. The measurements were conducted at room temperature using DCM as the solvent. The concentration of the SAM sample was 5×10^-4^ M, with 0.1 M tetrabutylammonium hexafluorophosphate (n-Bu_4_NPF_6_) as the supporting electrolyte. Prior to testing, the DCM solution was purged with argon for 5 minutes to remove dissolved oxygen. The scan rate was set to 50 mV/s. The trap density (*N*_trap_) can be calculated through space-charge-limited current measurements by the equation:

$\text{N}_{\text{trap}}\text{=2}\text{ε∙}\text{ε}_{\text{0}}\text{∙​}\text{V}_{\text{TFL}}\text{/}\text{q}\text{L}^{\text{2}}$ (3)

where $\text{ε}_{\text{0}}$ is the vacuum permittivity, $\text{ε}$ is the relative dielectric constant, $\text{​}\text{V}_{\text{TFL}}$ is the trap-filled limit voltage, $\text{q}$ is the elementary charge, and $\text{L}$ is the perovskite thickness. The surface coverage of SAM molecules on FTO substrates were determined by cyclic voltammetry using a three-electrode system. The working electrode was either bare FTO or FTO modified with the SAM molecules. A platinum sheet was used as the counter electrode, and Ag/AgCl electrode was used as the reference electrode. The electrolyte consisted of 0.1 M tetrabutylammonium hexafluorophosphate (n-Bu_4_NPF_6_) dissolved in o-dichlorobenzene (o-DCB). The scan rate was set to 10, 20, 30, 50, 70, and 100 mV/s respectively.

The thiol content was quantified using Ellman’s method. For the assay, 2 mL of the sample solution was mixed with 2 mL of 0.1% 5,5′-dithiobis(2-nitrobenzoic acid) (DTNB) in 0.2 M Tris–HCl buffer (pH 8.0) and shaken for 5 min. The optical density of the resulting 2-nitro-5-thiobenzoate (TNB) was measured at 412 nm using a Multiskan FC microplate reader (Thermo Fisher Scientific).

Current density-voltage (*J-V*) characteristics of devices were measured under AM 1.5 G illumination using a solar simulator equipped with a Keithley 2400 source meter and a 450W Xenon lamp (Class 3A, 94023A, Newport) calibrated light intensity by a reference silicon solar cell (91150V). Incident photon-to-current efficiency (IPCE) values were measured on a computer-controlled IPCE system (Newport 66902) equipped with a 100W xenon lamp, a monochromator, and a Keithley 2400 source meter, and collected in DC mode. Both the *J-V* measurements and IPCE tests were performed under ambient conditions (~25 °C, ~50±10% RH). For operation stability testing, the devices were placed in ambient air at 50±10% RH. Meanwhile, a fixed bias voltage equivalent to the initial maximum power point (MPP) voltage was continuously applied, with illumination provided by an AM 1.5 G solar simulator without UV filtering.

Broadband sum frequency generation (BB-SFG) spectroscopy was performed using the following setup. The output of a Ti:Sapphire femtosecond laser (Legend Elite Duo Femto, Coherent Inc.) was used to pump two optical parametric amplifiers (Light Conversion, TOPAS-C and TOPAS-400) to create the broadband femtosecond infrared pulse (IR, 150 fs, 160 cm^-1^ FWHM) and a narrowband picosecond visible pulse (VIS, 2.5 ps, 7 cm^-1^ FWHM). Here, the VIS (760 nm, 4 μJ/pulse) and IR (3500 nm, 8 μJ/pulse) temporally and spatially overlapped on the sample surface at ~60° incident angle. The time delay between the VIS and IR pulses was controlled by a motorized delay stage. Time zero reference between the VIS and IR was set to maximize the SFG intensity from the FTO electrode surface with the IR and VIS pulses overlapped. And positive time indicated the IR pulse arrived at the sample prior to the VIS pulse. All the BB-SFG signals were taken in the ppp polarization combination (p-polarized SFG, VIS, and IR), and recorded by a spectrograph (Princeton, Acton SP2500) with a CCD camera (Princeton, PIXIS 100BR). SFG spectra were collected with 60 s integration times.

The perovskite layer used for characterization was fabricated using a precursor solution with a composition of Cs_0.05_MA_0.15_FA_0.80_Pb(I_0.85_Br_0.15_)_3_ at a concentration of 1.3 M, prepared in a mixed solvent of DMF, DMSO, and NMP (volume ratio 4:0.9:0.1), with 0.3 mol% BMIMBF_4_ added as an additive. The solution was stirred overnight at room temperature and filtered through a 0.45 μm PTFE syringe filter prior to use. The films were deposited by spin-coating at 1500 rpm for 5 s followed by 6000 rpm for 30 s, during which chlorobenzene was dropped onto the substrate 5 s before the end of the second step. The resulting films were then annealed at 100 °C for 30 minutes.

Computational Methods

Geometry optimization and dipole moment calculations were performed using density functional theory (DFT) at the B3LYP/def2-SVP level with Grimme’s D3(BJ) dispersion correction, as implemented in the Gaussian 16 software package. HOMO–LUMO energy levels and molecular orbitals were analyzed using Multiwfn 3.8 (dev) based on formatted checkpoint files, and visualized with VMD 1.9.3. Electrostatic potential maps were generated from the Gaussian output and visualized using GaussView.

Fabrication of Perovskite Solar Cells

The FTO substrates (7Ω per square, 2×2 cm^2^) were cleaned ultrasonically with detergent, deionized water, acetone, and ethanol. After drying with N_2_, each substrate was treated with ozone for 15 min. 2PACz and S-2PACz were dissolved in ethanol and chlorobenzene, respectively, both at a concentration of 0.3 mg/mL. The solutions were then spin-coated onto cleaned FTO substrates at 3000 rpm for 30 s, followed by annealing at 100 °C for 10 min. For the Cys-S-2PACz HTL, cysteine hydrochloride (0.2 mg/mL in water) was first spin-coated onto FTO at 2000 rpm for 20 s, followed by annealing at 100 °C for 10 min. After cooling to room temperature, the S-2PACz solution was spin-coated at 3000 rpm for 30 s, followed by a second annealing at 100 °C for 10 min. The perovskite composition was Rb_0.05_Cs_0.05_MA_0.05_FA_0.85_Pb(I_0.95_Br_0.05_)_3_. The perovskite precursor was prepared in a mixed solvent of DMF and DMSO (4:1, v/v) at a concentration of 1.5 M. The perovskite precursor was spin-coated at 3000 rpm for 40 s, with chlorobenzene spraying at 8 s before the end of the process. Then the film was annealed at 100 °C for 30 min. After cooling to room temperature, 50 μL of EDAI_2_ solution (0.3 mg/mL in IPA) was spin-coated onto the perovskite surface at 3000 rpm for 30 s, followed by an additional annealing step at 100 °C for 5 min. Afterward, PCBM (20 mg/mL in CB) was spin-coated on the film at 2000 rpm for 30 s. After that, BCP (0.5 mg/mL in IPA) was spin-coated at 4000 rpm for 30 s. Finally, a 100 nm Ag electrode was thermally evaporated under high vacuum (< 1×10^-7^ Torr). The effective area of one cell was 0.12 cm^2^.

Fabrication of Perovskite Solar Modules

First, the patterned FTO glass substrates with the scribed P1 lines were cleaned in the same manner as for small-area PSCs. The HTL was blade-coated onto the substrate using the same concentration and annealing conditions as employed for the small-area PSCs. The precursor solution was prepared by dissolving 13.0 mg CsI, 230.5 mg PbI_2_, 77.4 mg FAI, 13.8 mg PbCl_2_ and 10.0 mg MACl in a mixed anhydrous solvent of DMF and NMP (293 μL and 48 μL, respectively). The perovskite precursor solution was stirred for 3 h and subsequently filtered through a 0.45 µm PTFE membrane before blade coating. The perovskite precursor was blade-coated and subsequently annealed at 100 °C for 30 min. All blade-coating procedures were performed under ambient conditions. Afterward, C_60_ (20 nm) and BCP (6 nm) were sequentially deposited in a vacuum chamber at 5×10^-4^ Pa. Before Cu deposition, P2 lines were patterned using a pulsed laser with a pulse energy of 30 μJ and a pulse frequency of 30 kHz to expose the bottom FTO electrode. Finally, a 100 nm Cu layer was thermally evaporated as the counter electrode, and each sub-cell was separated by laser scribing to form P3 lines.

Module Encapsulation

The excess edge of the large-area perovskite film was etched by laser scribing first. The encapsulant polyisobutylene and cover glass were sequentially laminated onto the module. Then the as-prepared device was transferred to a vacuum hot-press machine to bond the module with the cover glass, and the hot-press process was maintained at 110 °C for 10 min.

Note on Perovskite Composition Used for Characterization vs. Device Fabrication

Surface and interfacial characterizations were initially conducted using a perovskite composition of Cs_0.05_MA_0.15_FA_0.80_Pb(I_0.85_Br_0.15_)_3_ (referred to as Perovskite A). To improve device performance, a slightly modified composition, Rb_0.05_Cs_0.05_MA_0.05_FA_0.85_Pb(I_0.95_Br_0.05_)_3_ (Perovskite B), was later adopted for final device fabrication. Importantly, the HTM-related interfacial phenomena discussed in this work are expected to remain largely unaffected by this compositional shift. We therefore consider the interfacial characterizations conducted on Perovskite A to be representative of the HTM-perovskite interactions occurring in the final devices.

Synthesis of S-2PACz

**Figure S1.** Synthesis of S-2PACz.

Synthesis of Compound I

A mixture of 4-bromothioanisole (1.072 g, 5.28 mmol), 1,1'-bis(diphenylphosphino)ferrocene (dppf, 0.349 g, 0.63 mmol), Pd(dppf)Cl_2_·CH_2_Cl_2_ (0.021 g, 0.0275 mmol), and sodium tert-butoxide (NaOt-Bu, 0.697 g, 7.25 mmol) was added to a 25 mL round-bottom two-necked flask. The flask was evacuated and backfilled with argon three times using an oil-pump vacuum. Subsequently, 4-(methylthio)aniline (0.881 g, 6.32 mmol) and anhydrous 1,4-dioxane (15 mL) were added via syringe under argon atmosphere. The reaction mixture was stirred and refluxed at 120 °C for 24 h. After cooling to room temperature, the reaction mixture was extracted with water (30 mL) and ethyl acetate (3 × 30 mL). The combined organic layers were dried over anhydrous Na_2_SO_4_, and the solvent was removed under reduced pressure. The crude product was purified by column chromatography on silica gel (300–400 mesh) using petroleum ether/ethyl acetate (30:1, v/v) as eluent to afford compound **I** as a yellowish-brown solid (93% yield).

^1^H NMR (400 MHz, CDCl_3_, δ, ppm): 7.23–7.21 (m, 4H, ArH), 6.97–6.95 (m, 4H, ArH), 5.65 (s, 1H, NH), 2.44 (s, 6H, SCH_3_). ^13^C NMR (100 MHz, CDCl_3_, δ, ppm): 141.24, 129.85, 129.25, 118.54, 17.83.


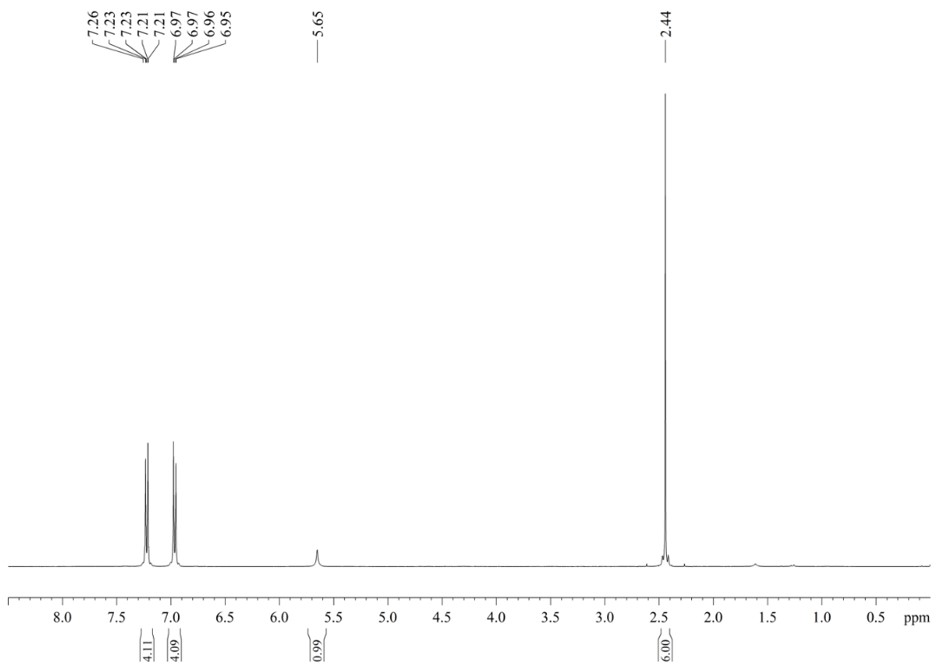


**Figure S2.** ^1^H NMR spectrum of **Compound I**.


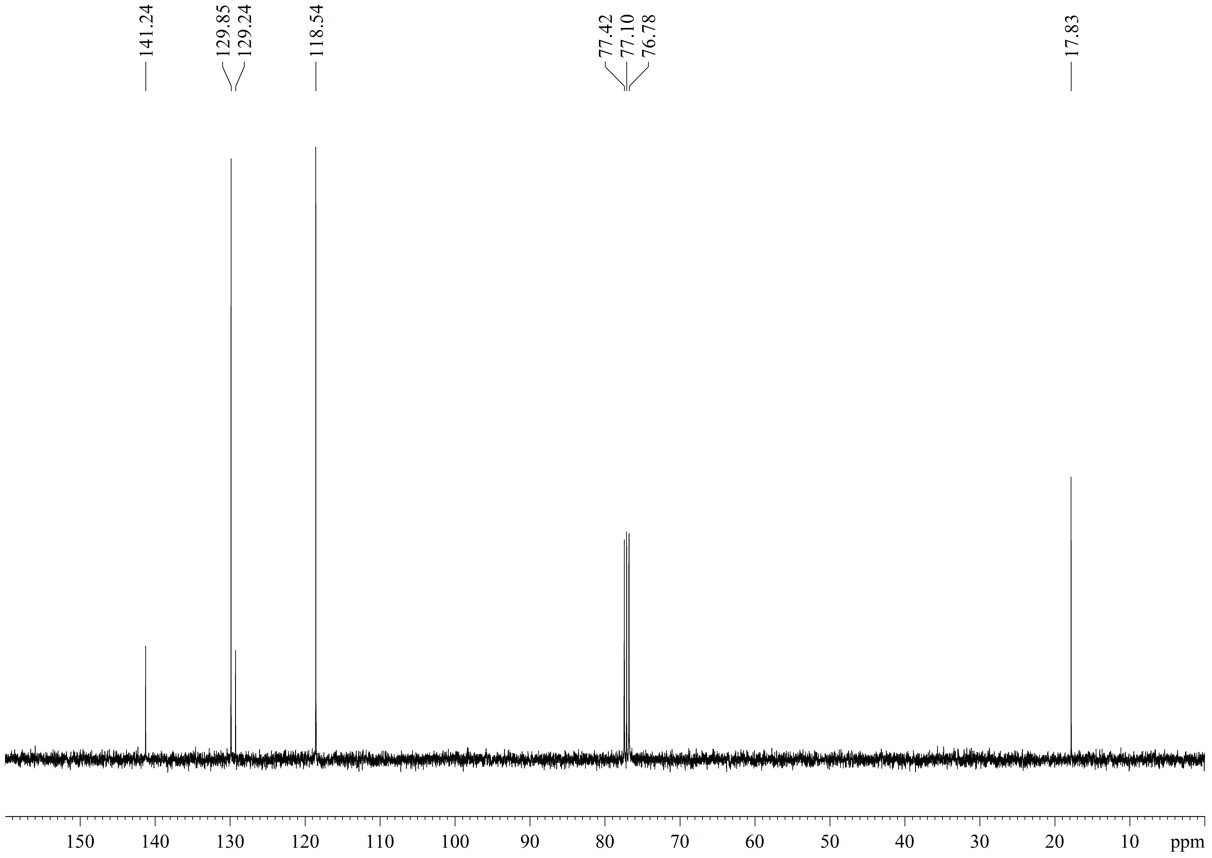


**Figure S3.** ^13^C NMR spectrum of **Compound I**.

**Synthesis of Compound II**

3,6-Dibromocarbazole (3.25 g, 10 mmol) and 1,2-dibromoethane (30 mL) were added to a 150 mL round-bottomed flask and heated at 60 °C for 72 h. At 0, 24, and 48 h, a solution of tetrabutylammonium bromide (0.323 g, 1 mmol) and 50 wt% aqueous KOH (2.8 mL, 50 mmol) was added to the reaction mixture. Upon completion, the mixture was extracted with water (60 mL) and dichloromethane (3 × 60 mL). The combined organic layers were dried over anhydrous Na_2_SO_4_ and concentrated under reduced pressure. The crude product was purified by column chromatography on silica gel (300–400 mesh) using petroleum ether/ethyl acetate (15:1, v/v) as the eluent to afford compound **II** as a white solid (92% yield).

^1^H NMR (400 MHz, CDCl_3_, δ, ppm): 8.12 (d, *J* = 1.7 Hz, 2H, ArH), 7.57 (dd, *J* = 8.7, 1.8 Hz, 2H, ArH), 7.29 (d, *J* = 8.7 Hz, 2H, ArH), 4.64 (t, *J* = 7.2 Hz, 2H, –CH_2_–Br), 3.65 (t, *J* = 7.2 Hz, 2H, –CH_2_–N). ^13^C NMR (100 MHz, CDCl_3_, δ, ppm): 138.94, 129.37, 123.77, 123.47, 112.80, 110.22, 44.86, 27.89.


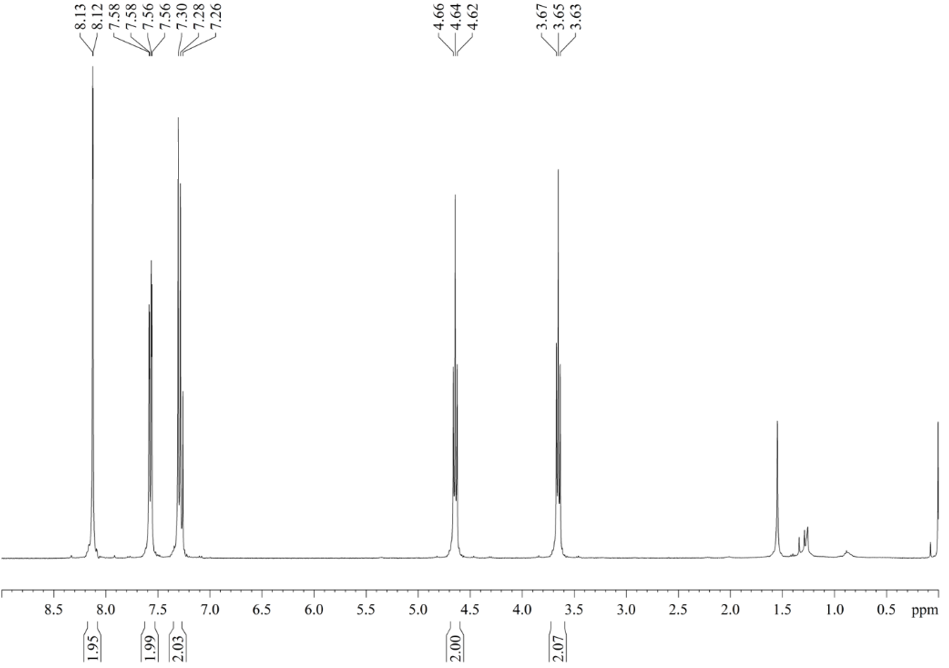


**Figure S4.** ^1^H NMR spectrum of **Compound II**.


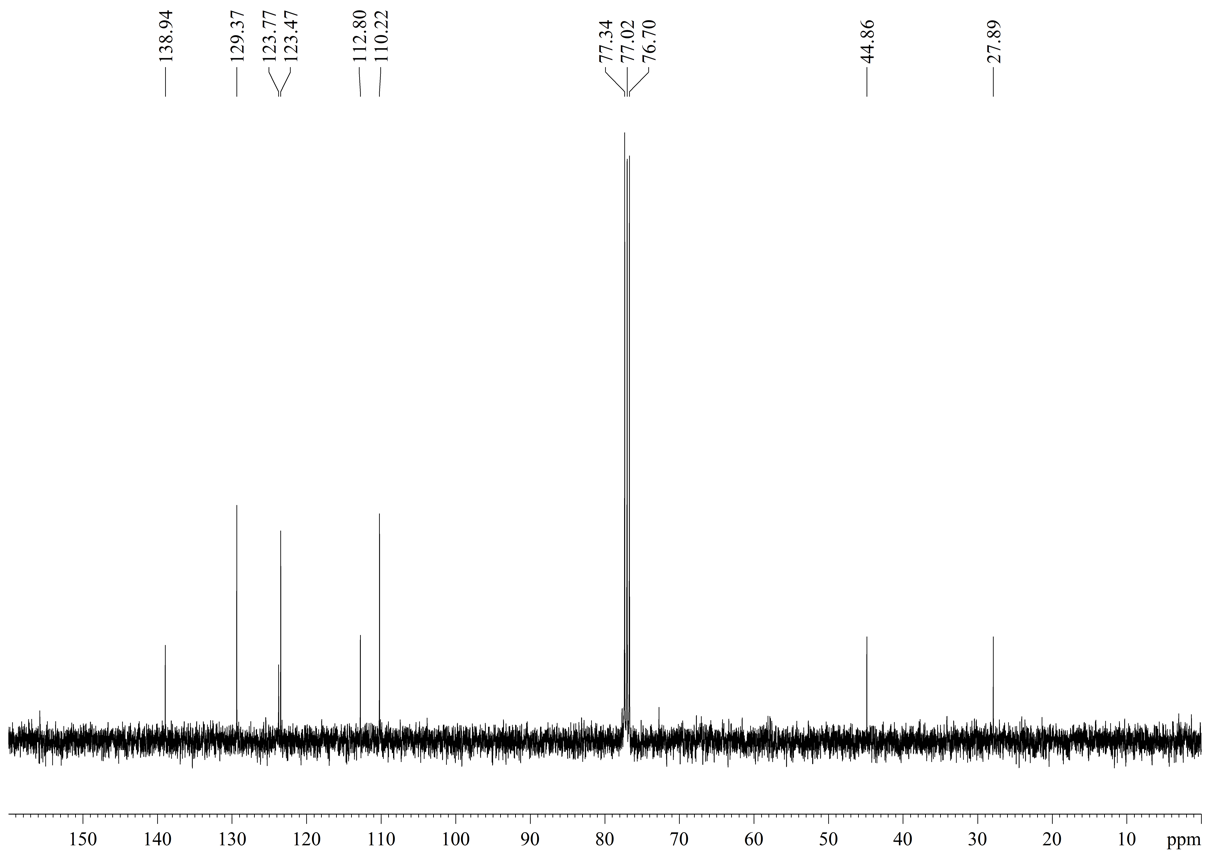


**Figure S5.** ^13^C NMR spectrum of **Compound II**.

**Synthesis of Compound III**

A mixture of compound **II** (346 mg, 0.8 mmol), compound **I** (420 mg, 1.6 mmol), Pd_2_(dba)_3_ (15 mg, 0.016 mmol), P(t-Bu)_3_·HBF_4_ (7 mg, 0.024 mmol), and sodium tert-butoxide (NaOt-Bu, 230 mg, 2.4 mmol) was added to a 25 mL two-necked round-bottom flask under argon atmosphere. The flask was evacuated and backfilled with argon three times using an oil-pump vacuum. Subsequently, anhydrous toluene (8 mL), pre-dried over calcium hydride and purged with argon, was added via syringe. The reaction mixture was stirred under reflux at 110 °C for 24 h. After completion, the mixture was filtered through a Celite® pad to remove viscous byproducts and insoluble materials. The filter cake was rinsed with ethyl acetate several times. The combined filtrate was concentrated under reduced pressure, then extracted with water (20 mL) and ethyl acetate (3 × 20 mL). The organic layer was dried over anhydrous Na_2_SO_4_ and evaporated under vacuum. The crude product was purified by column chromatography on silica gel (300–400 mesh) using petroleum ether/ethyl acetate (20:1, v/v) as eluent to yield compound **III** as a pale green solid (77% yield).

^1^H NMR (400 MHz, CDCl_3_, δ, ppm): 7.70 (d, *J* = 1.8 Hz, 2H, ArH), 7.34 (d, *J* = 8.7 Hz, 2H, ArH), 7.24 (d, *J* = 8.7 Hz, 2H, ArH), 7.15 (d, *J* = 8.7 Hz, 8H, ArH), 6.97 (d, *J* = 8.7 Hz, 8H, ArH), 4.67 (t, *J* = 7.2 Hz, 2H, –CH_2_Br), 3.70 (t, *J* = 7.2 Hz, 2H, –CH_2_–N), 2.45 (s, 12H, –SCH_3_). ^13^C NMR (100 MHz, CDCl_3_, δ, ppm): 146.26, 139.94, 137.70, 130.35, 128.93, 125.67, 123.77, 123.25, 118.68, 109.63, 44.98, 28.25, 17.23.


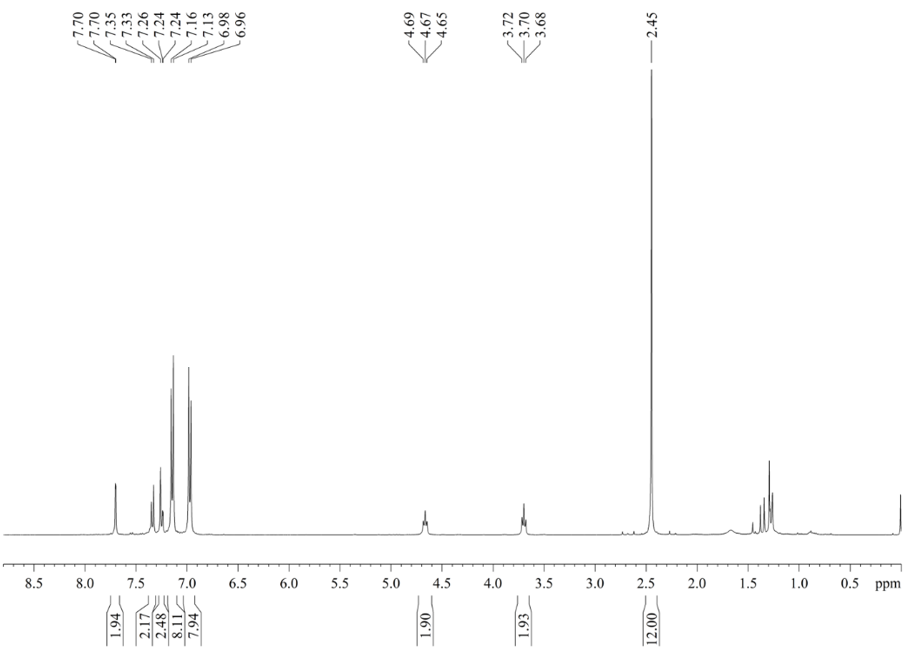


**Figure S6.** ^1^H NMR spectrum of **Compound III**.


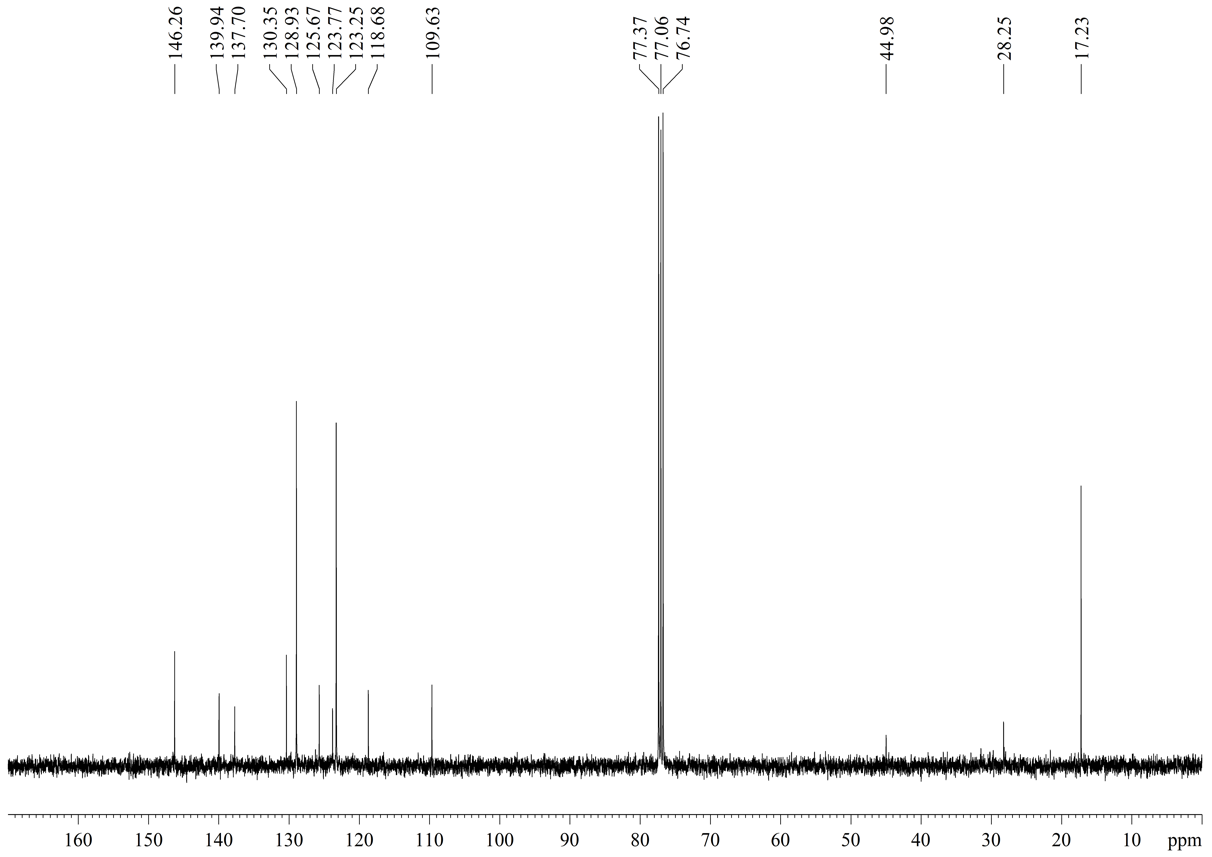


**Figure S7.** ^13^C NMR spectrum of **Compound III**.

**Synthesis of Compound IV**

Compound **III** (306 mg, 0.386 mmol) and triethyl phosphite (2.5 mL) were added sequentially to a 50 mL round-bottomed flask. The reaction mixture was stirred and refluxed at 165 °C under an argon atmosphere for 18 h. After cooling to room temperature, the solvent and excess triethyl phosphite were removed under reduced pressure to afford a yellow oil-like residue. The crude product was subjected to flash column chromatography on silica gel (300–400 mesh), first eluted with petroleum ether/ethyl acetate (1:2, v/v), followed by n-hexane/acetone (3:1, v/v). Compound **IV** was obtained as a green solid in 70% yield.

^1^H NMR (500 MHz, DMSO‑*d*_6_, δ, ppm): 7.88 (d, *J* = 2.0 Hz, 2H, ArH), 7.56 (d, *J* = 8.8 Hz, 2H, ArH), 7.20 (dd, *J* = 8.7, 2.1 Hz, 2H, ArH), 7.15–7.12 (m, 8H, ArH), 6.87–6.85 (m, 8H, ArH), 4.58–4.53 (m, 2H, –CH_2_–N), 3.95– 3.89 (m, 4H, –CH_2_–O), 2.39 (s, 12H, –SCH_3_), 2.34–2.27 (m, 2H, –CH_2_–P), 1.12 (t, *J* = 7.0 Hz, 6H, –CH_3_). ^13^C NMR (125 MHz, DMSO‑*d*_6_, δ, ppm): 146.14, 139.14, 138.00, 130.20, 128.68, 126.10, 123.60, 123.02, 119.52, 111.17, 61.68, 61.63, 37.33, 25.45, 24.36, 16.59, 16.54, 16.41. ^31^P NMR (202 MHz, DMSO‑*d*_6_, δ, ppm): 27.82.


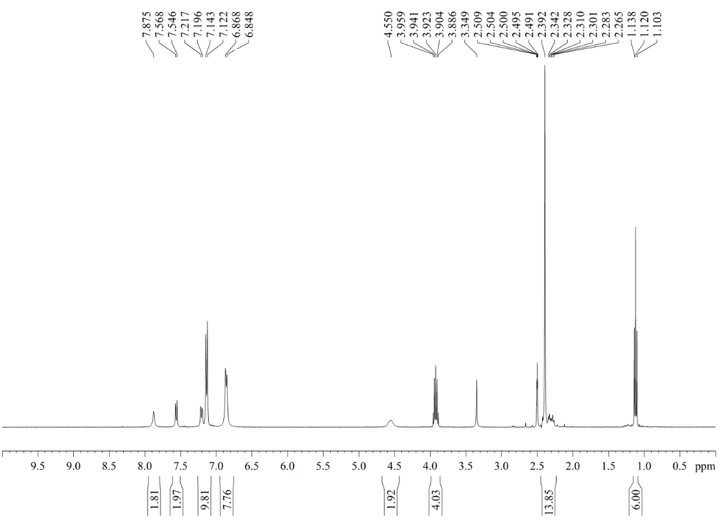


**Figure S8.** ^1^H NMR spectrum of **Compound IV**.


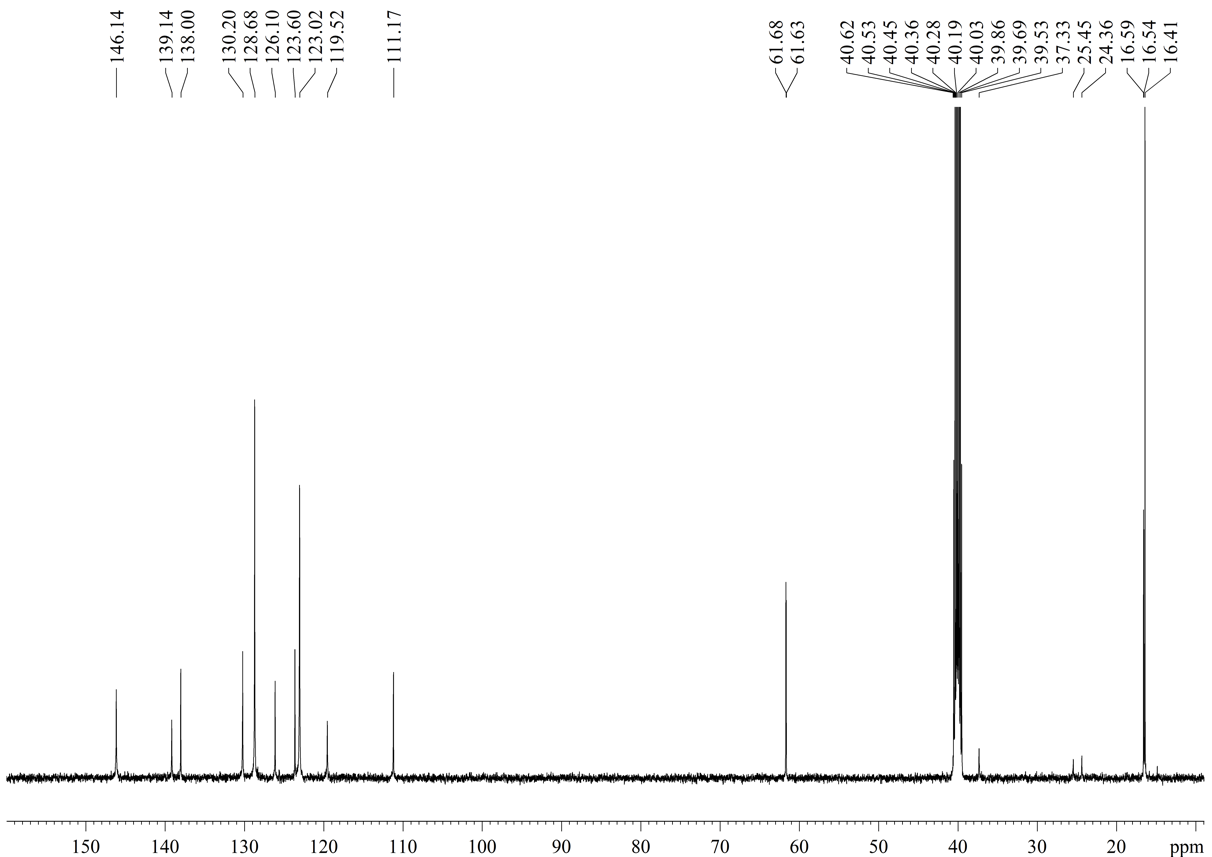


**Figure S9.** ^13^C NMR spectrum of **Compound IV**.

**Synthesis of S-2PACz**

Compound **IV** (193 mg, 0.22 mmol) was added to a 25 mL two-necked round-bottomed flask. The flask was evacuated and backfilled with argon several times. Ultra-dry 1,4-dioxane (6 mL) was added via syringe to dissolve the solid completely, followed by dropwise addition of trimethylsilyl bromide (TMSBr, 0.45 mL, 3.3 mmol, 15 eq.). The reaction mixture was stirred at 35 °C under argon for 48 h. After completion, the reaction solution was transferred to a 50 mL single-neck round-bottomed flask and concentrated under reduced pressure to remove the solvent and excess TMSBr. The residue was then treated with methanol (5 mL) and deionized water (5 mL) and stirred at 35 °C for 38 h. The resulting solid was collected by filtration, washed thoroughly with water, and dried under vacuum at 60 °C for 12 h to afford **S-2PACz** as a green solid (90% yield).

^1^H NMR (400 MHz, DMSO‑*d*_6_, δ, ppm): 7.81 (d, *J* = 1.6 Hz, 2H, ArH), 7.49 (d, *J* = 8.7 Hz, 2H, ArH), 7.17–7.09 (m, 10H, ArH), 6.84 (d, *J* = 8.7 Hz, 8H, ArH), 4.52 (s, 2H, –CH_2_–N), 2.37 (s, 12H, –SCH_3_), 2.07–2.01 (m, 2H, –CH_2_–P). ^13^C NMR (100 MHz, DMSO‑*d*_6_, δ, ppm): 146.11, 139.11, 137.94, 130.16, 128.66, 126.25, 123.55, 123.01, 119.60, 110.86, 38.37, 29.51, 16.41. ^31^P NMR (162 MHz, DMSO‑*d*_6_, δ, ppm): 21.42. HRMS (ESI^+^): *m/z* [M + Na]^+^ calculated for C_42_H_40_N_3_O_3_PS_4_Na^+^: 816.1582; found: 816.1587.


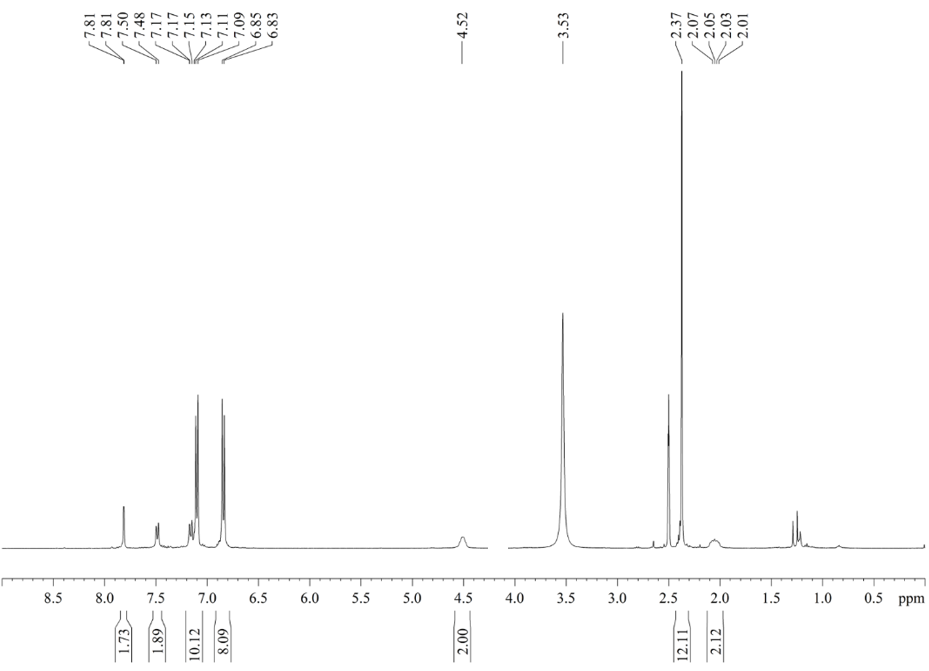


**Figure S10.** ^1^H NMR spectrum of **S-2PACz**.


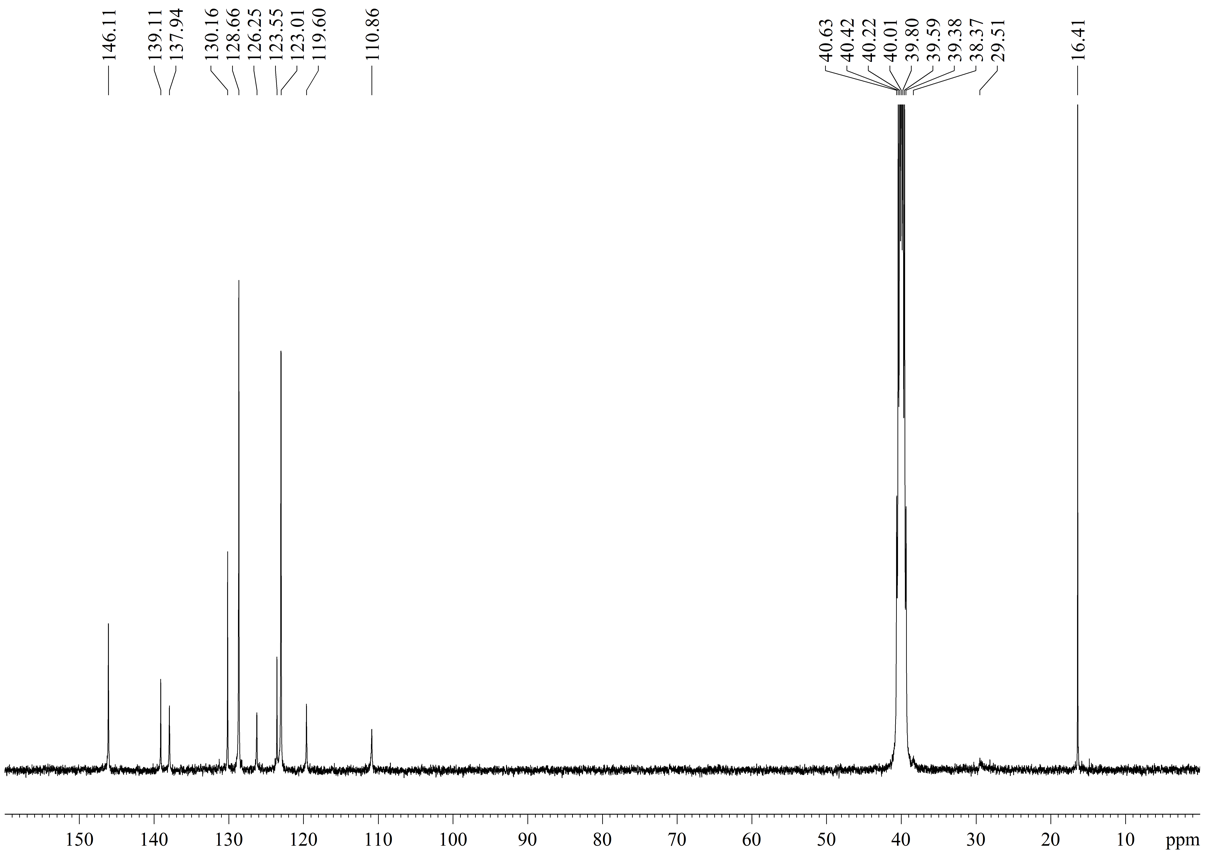


**Figure S11.** ^13^C NMR spectrum of **S-2PACz**.

**
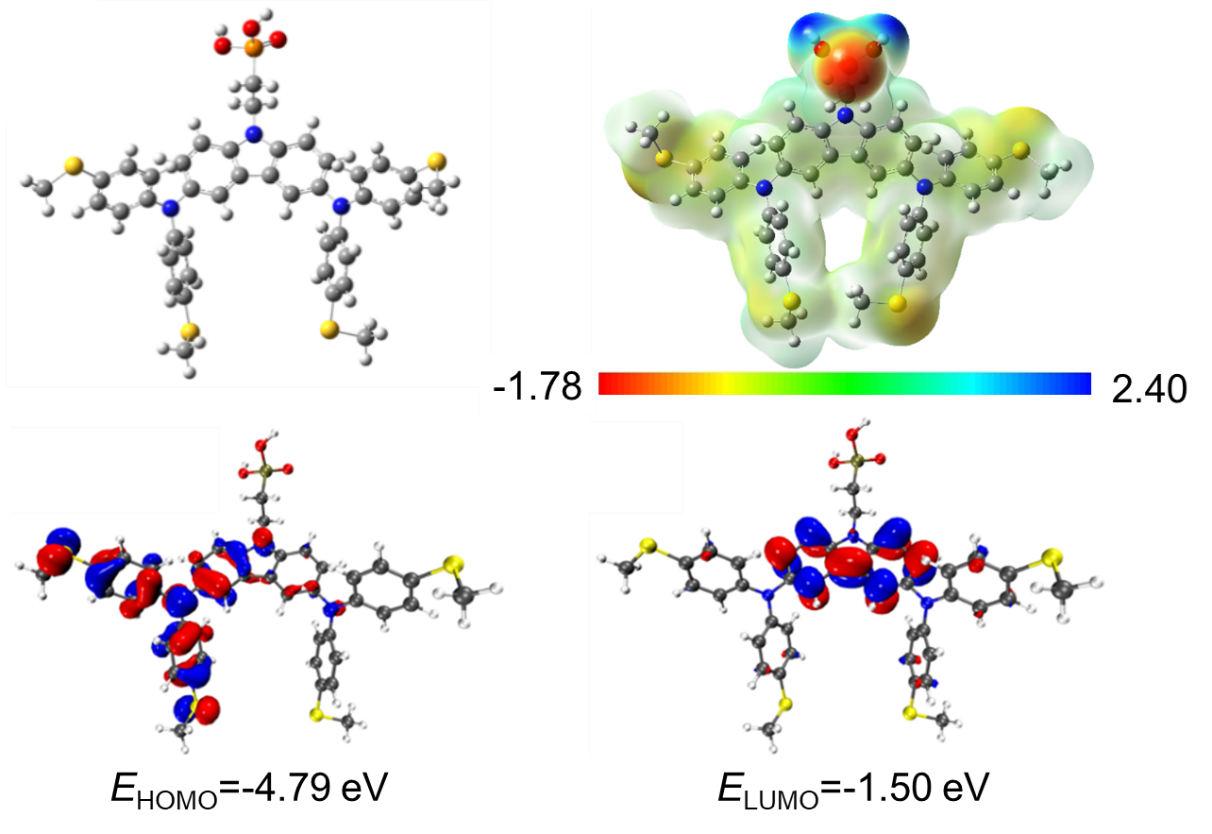
**

**Figure S12.** DFT optimized geometries, molecular surface electrostatic potential isosurface, and molecular frontier orbitals of S-2PACz.


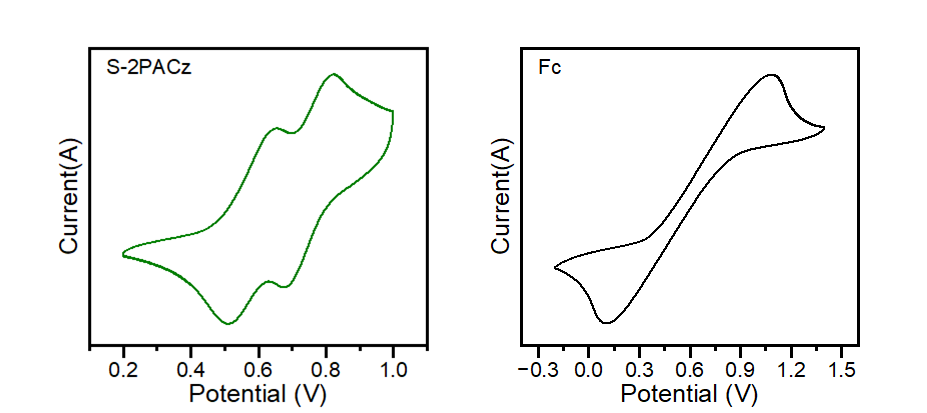


**Figure S13.** Cyclic voltammogram of S-2PACz (left) and ferrocene (right), with ferrocene used as external reference. All potentials are reported vs. $E_{1/2(Fc/\mathrm{Fc}^{+})}$.


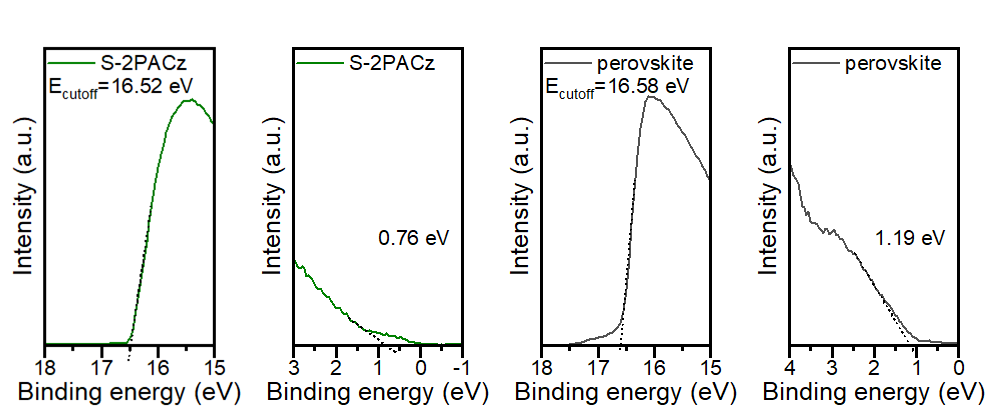


**Figure S14.** UPS results of S-2PACz/FTO and perovskite/FTO (perovskite composition: Cs_0.05_MA_0.15_FA_0.80_Pb(I_0.85_Br_0.15_)_3_.


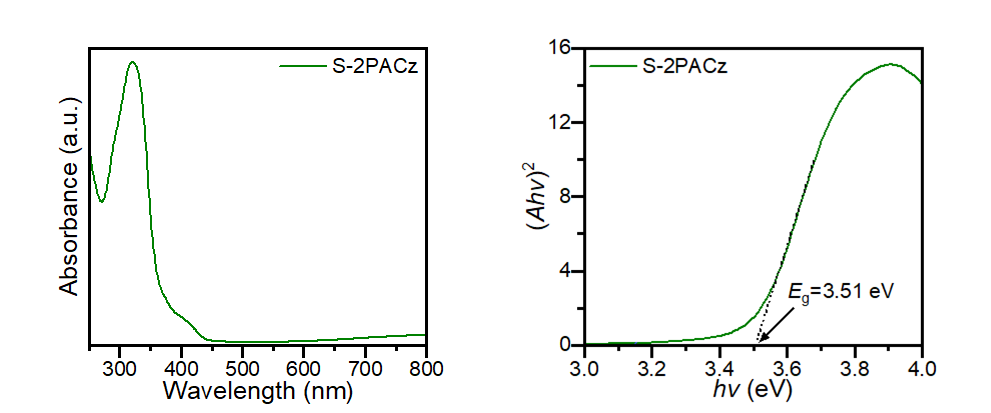


**Figure S15.** UV-vis absorption spectra of S-2PACz in solution (10^-5^ M in dichloromethane).


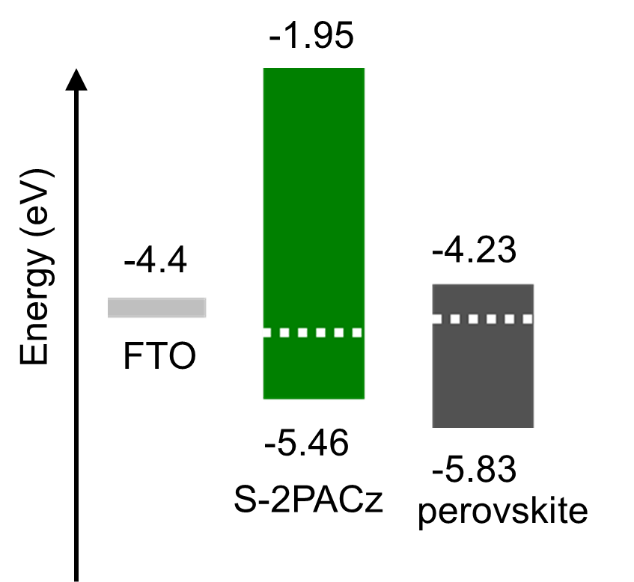


**Figure S16.** Energetic diagram of FTO/S-2PACz/perovskite (perovskite composition: Cs_0.05_MA_0.15_FA_0.80_Pb(I_0.85_Br_0.15_)_3_).


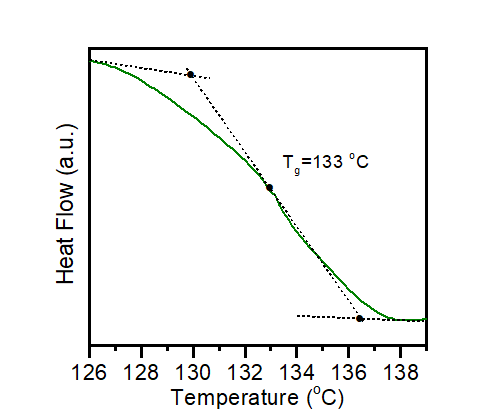


**Figure S17.** Differential scanning calorimetry thermogram of S-2PACz recorded at a heating rate of 10 ^o^C min^-1^.


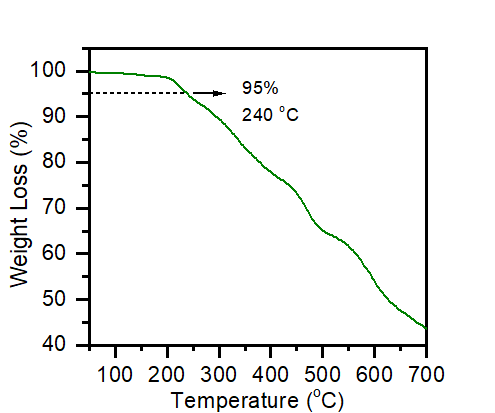


**Figure S18.** Thermogravimetric analysis of S-2PACz at heating rate of 10 ^o^C min^-1^ under N_2_ atmosphere.


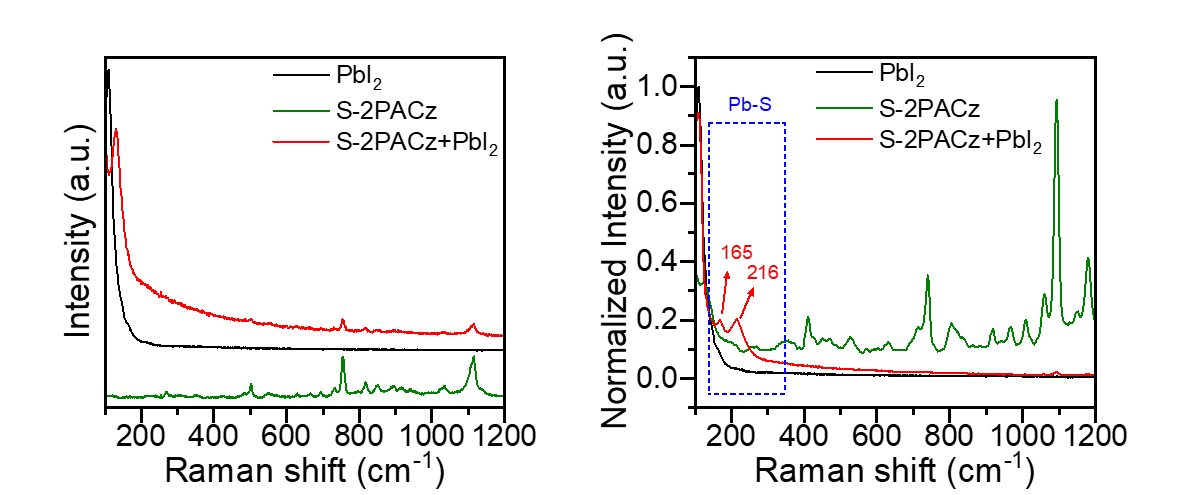


**Figure S19.** Raman spectra of PbI_2_, S-2PACz and their mixture (left: as-measured spectra; right: normalized spectra).


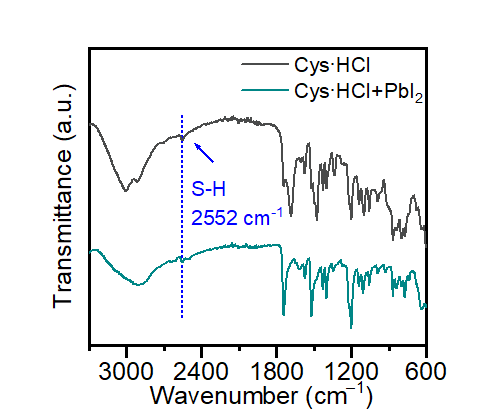


**Figure S20.** FTIR spectra of Cys·HCl and the Cys·HCl + PbI_2_ mixture.


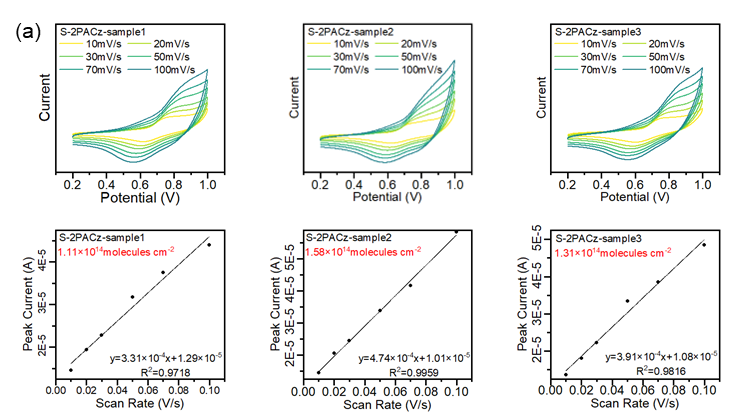


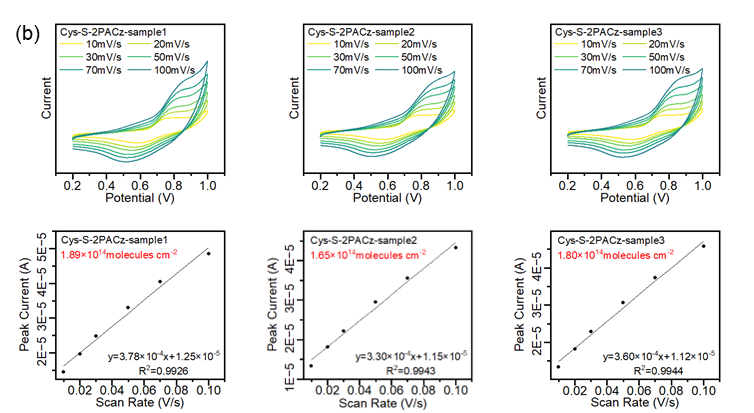


**Figure S21.** Cyclic voltammograms of the hole-transport material on FTO substrates measured in o-DCB solution under different scan rates and the corresponding plot of peak current versus scan rate.


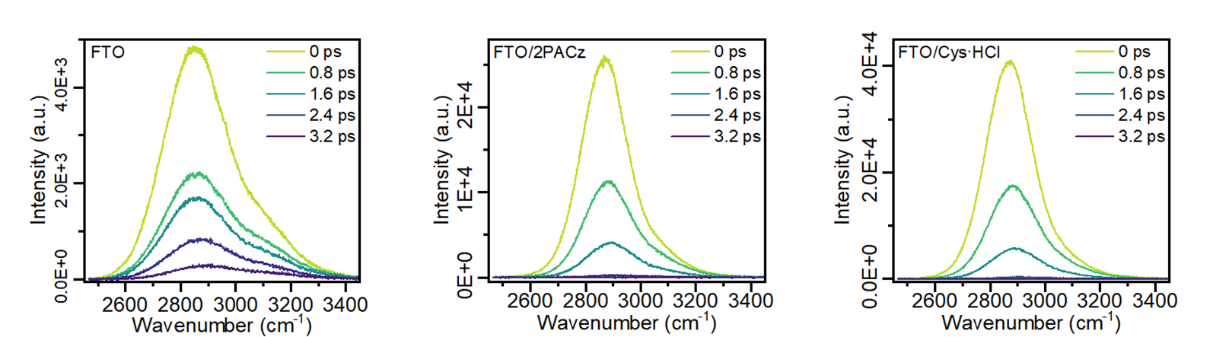


**Figure S22.** BB-SFG spectra of FTO, FTO/2PACz, and FTO/Cys·HCl measured at different delay times.


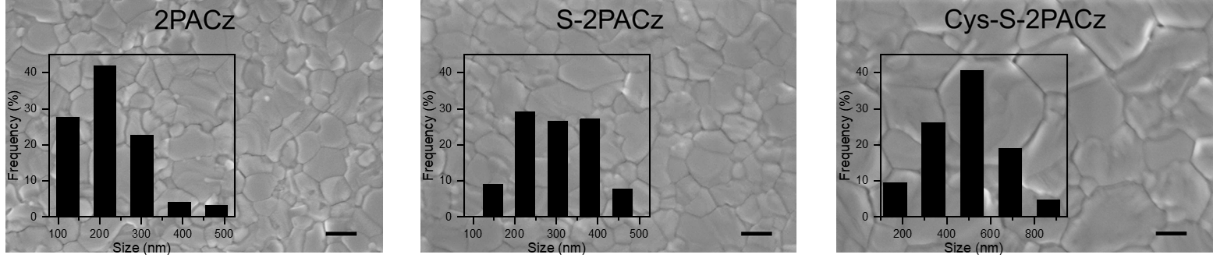


**Figure S23.** SEM images of the perovskite film on the 2PACz, S-2PACz and Cys-S-2PACz SAMs (top). The scale bar is 300 nm. (Inset: Particle size distribution of the perovskite films)


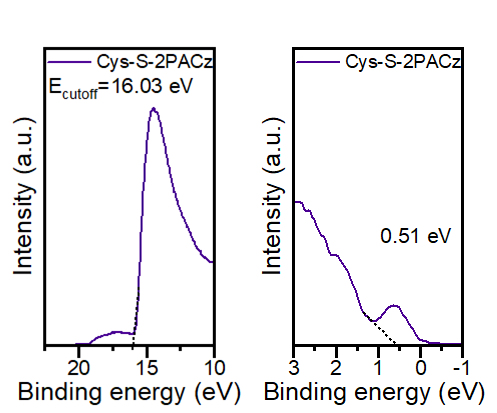


**Figure S24.** UPS spectra of Cys-S-2PACz deposited on FTO.


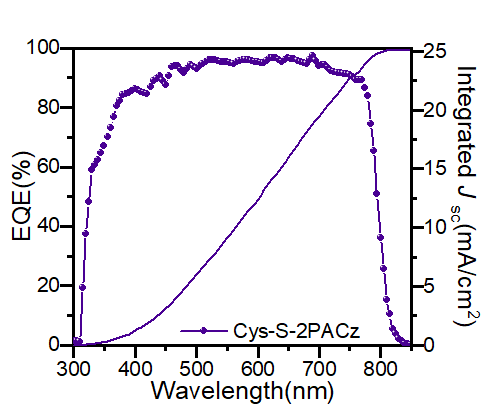


**Figure S25.** Incident photon-to-electron conversion efficiency spectrum and integrated current density of the Cys-S-2PACz-based perovskite solar cell.


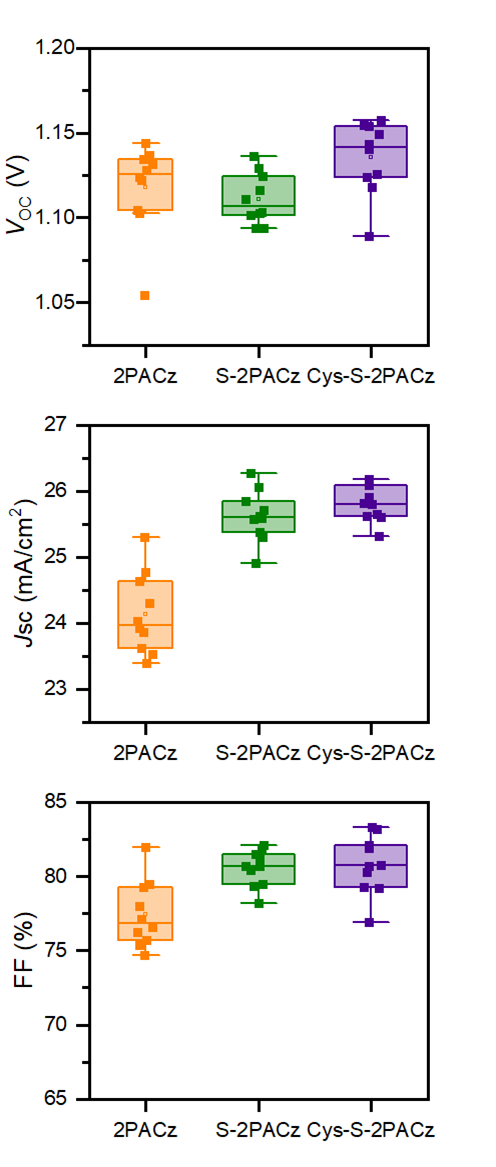


**Figure S26.** Photovoltaic parameter statistics of 10 individual devices fabricated with different SAMs.


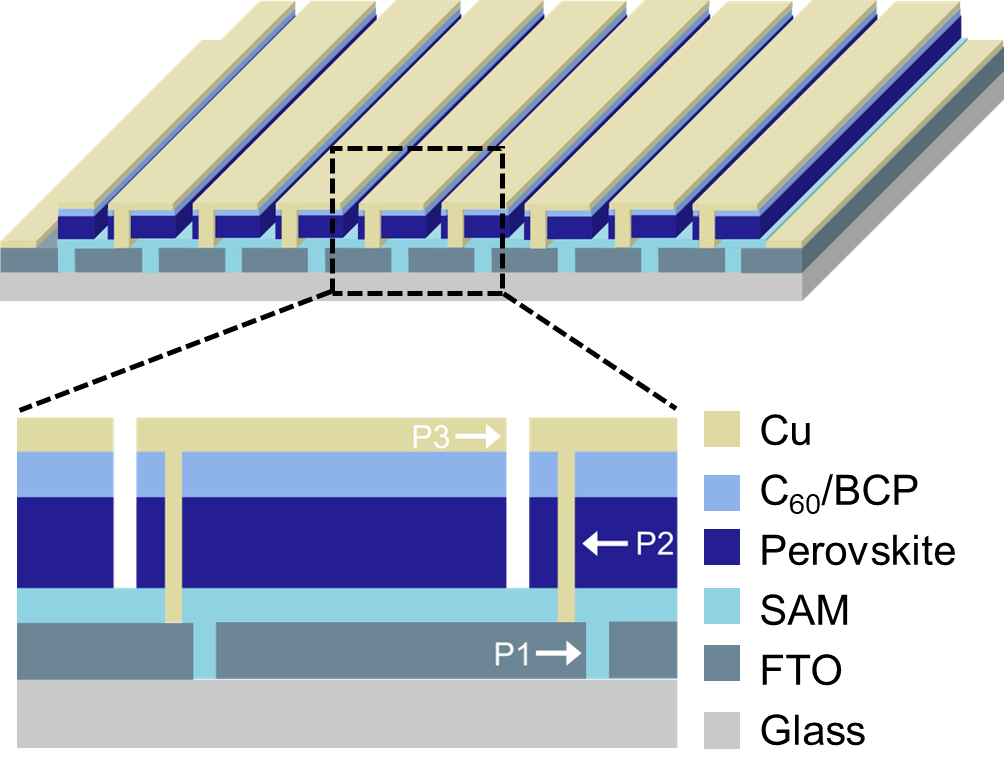


**Figure S27.** Schematic diagram of the module structure.


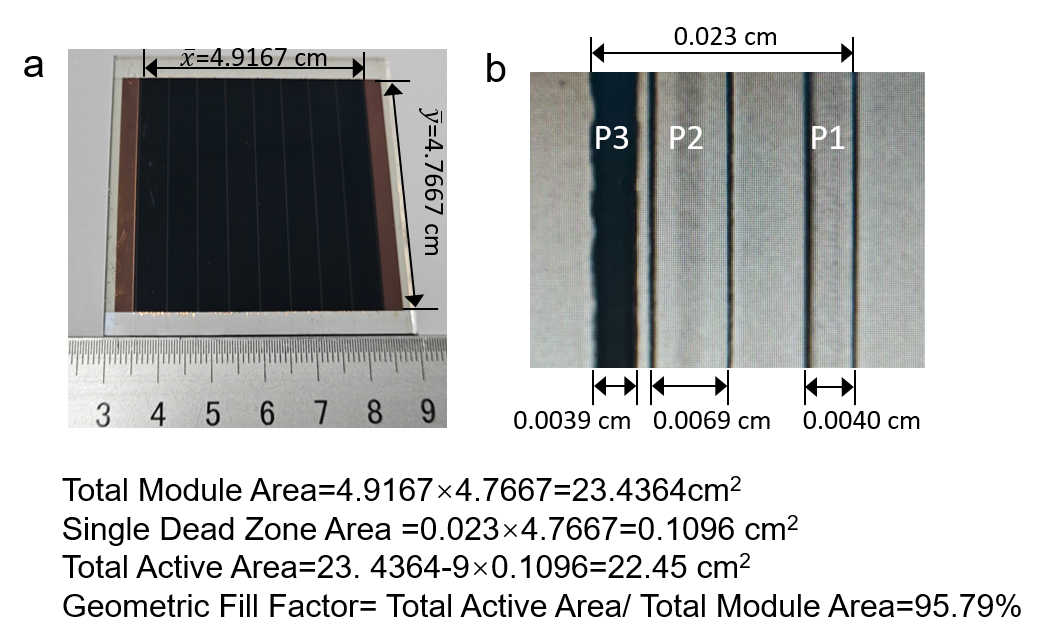


Total Module Area= 4.9167×4.7667=23.4364 cm^2^

Single Dead Zone Area= 0.023×4.7667=0.1096 cm^2^

Total Active Area= 23. 4364-9×0.1096=22.45 cm^2^

Geometric Fill Factor= Total Active Area/ Total Module Area=95.79%

**Figure S28.** (a) Photograph of the back side of the module. (b) Magnified view of channels P1, P2, and P3, showing the measured widths and the dead zone width. The corresponding calculations are provided below the figure.


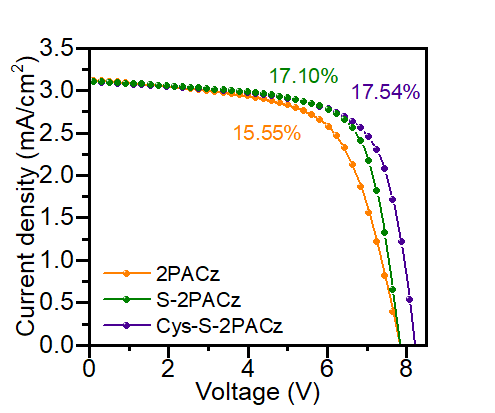


**Figure S29.** *J–V* curves of the champion modules fabricated with different SAMs.


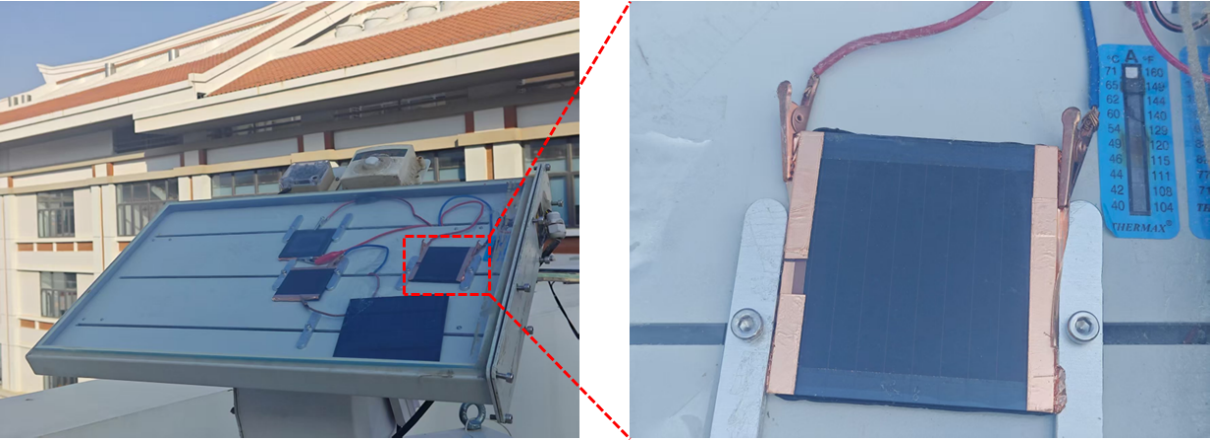


**Figure S30.** Photographs of the outdoor real-time power generation monitoring system with automatic sun-tracking (inset: maximum surface temperature measured using thermochromic paper). This system employs a light intensity detector to automatically adjust the azimuth and tilt angles, ensuring optimal incident light alignment. This enables real-time monitoring of device performance under diurnal cycles, temperature variations, and varying weather conditions. The system was deployed outdoors in the Xiang’an campus of Xiamen University (24.4 °N, 118.2 ^°^E). Historical weather data are available at https://www.timeanddate.com/weather/china/xiamen/historic.

**Table S1.** Fitting parameters for the TRPL of perovskite films with various SAMs.

| Type | *B*_1_ (%) | *B*_2_ (%) | *𝜏*_1_ (ns) | *𝜏*_2_ (ns) | *𝜏*_ave_ (ns) |
| --- | --- | --- | --- | --- | --- |
| perovskite | 0.57 | 0.43 | 5.29 | 18.12 | 10.76 |
| S-2PACz | 0.73 | 0.27 | 1.69 | 5.21 | 2.63 |
| Cys-S-2PACz | 0.69 | 0.31 | 1.45 | 3.80 | 2.18 |

**Table S2.** Photovoltaic parameters of 2PACz, S-2PACz and Cys-S-2PACz-treated PSCs with an active area of 0.12 cm^2^.

| Device | Scan direction | *V*_OC_/V | *J*_SC_/mA^.^cm^-2^ | FF/% | PCE/% |
| --- | --- | --- | --- | --- | --- |
| 2PACz | Reverse | 1.14 | 25.26 | 75.22 | 21.64 |
|  | Forward | 1.12 | 25.06 | 74.95 | 21.12 |
| S-2PACz | Reverse | 1.14 | 25.91 | 80.68 | 23.84 |
|  | Forward | 1.13 | 26.06 | 78.21 | 23.02 |
| Cys-S-2PACz | Reverse | 1.15 | 26.09 | 82.10 | 24.72 |
|  | Forward | 1.14 | 26.18 | 76.91 | 23.01 |

**Table S3.** Photovoltaic parameters of 2PACz, S-2PACz and Cys-S-2PACz-treated PSCs with an active area of 22.45 cm^2^.

| Device | Scan direction | *V*_OC_/V | *J*_SC_/mA^.^cm^-2^ | FF/% | PCE/% |
| --- | --- | --- | --- | --- | --- |
| 2PACz | Reverse | 7.83 | 3.12 | 63.60 | 15.55 |
|  | Forward | 7.82 | 3.14 | 61.81 | 15.19 |
| S-2PACz | Reverse | 7.82 | 3.10 | 70.49 | 17.10 |
|  | Forward | 8.11 | 3.11 | 66.93 | 16.88 |
| Cys-S-2PACz | Reverse | 8.19 | 3.10 | 69.09 | 17.54 |
|  | Forward | 8.18 | 3.11 | 67.69 | 17.22 |

**Reference:**

[S1] C. Li, Y. Chen, Y. Li, Z. Zhang, J. Yang, Y. Wang, L. Gong, Z. Yuan, L. Liang, S. Liu, Y. Zhu, C. Lian, M. Haider, T. Guo, X. Xu, D. Li, E. Bi, P. Gao, *Angew. Chem. Int. Ed.* **2025**, *64*, e202502730.
